# Transcriptional Regulation of Wnt/β-Catenin Pathway in Colorectal Cancer

**DOI:** 10.3390/cells9092125

**Published:** 2020-09-19

**Authors:** Jia Bian, Marius Dannappel, Chunhua Wan, Ron Firestein

**Affiliations:** 1 Centre for Cancer Research, Hudson Institute of Medical Research, Clayton, VIC 3168, Australia; jia.bian@monash.edu (J.B.); marius.dannappel@hudson.org.au (M.D.); Chunhua.Wan@monash.edu (C.W.); 2 Department of Molecular and Translational Science, Monash University, Clayton, VIC 3800, Australia

**Keywords:** Wnt/β-catenin signaling pathway, transcriptional regulation, epigenetic regulation, colorectal cancer

## Abstract

The Wnt/β-catenin signaling pathway exerts integral roles in embryogenesis and adult homeostasis. Aberrant activation of the pathway is implicated in growth-associated diseases and cancers, especially as a key driver in the initiation and progression of colorectal cancer (CRC). Loss or inactivation of Adenomatous polyposis coli (APC) results in constitutive activation of Wnt/β-catenin signaling, which is considered as an initiating event in the development of CRC. Increased Wnt/β-catenin signaling is observed in virtually all CRC patients, underscoring the importance of this pathway for therapeutic intervention. Prior studies have deciphered the regulatory networks required for the cytoplasmic stabilisation or degradation of the Wnt pathway effector, β-catenin. However, the mechanism whereby nuclear β-catenin drives or inhibits expression of Wnt target genes is more diverse and less well characterised. Here, we describe a brief synopsis of the core canonical Wnt pathway components, set the spotlight on nuclear mediators and highlight the emerging role of chromatin regulators as modulators of β-catenin-dependent transcription activity and oncogenic output.

## 1. Introduction

**Figure 1:**
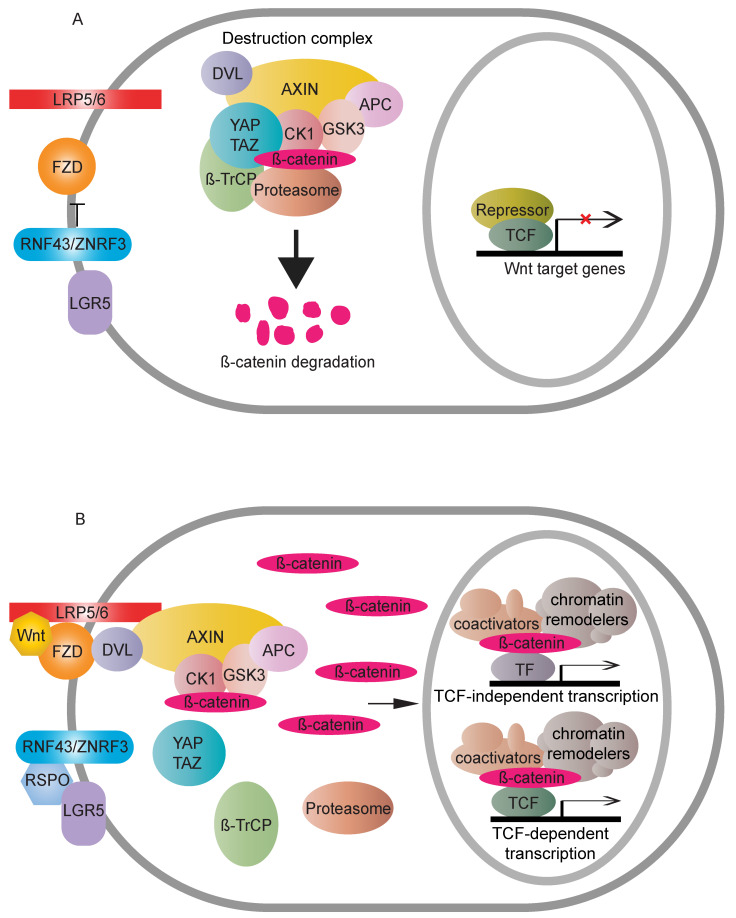
Overview of the Wnt canonical (β-catenin-dependent) pathway. (**A**) In the absence of a Wnt signal, cytoplasmic β-catenin is degraded by the destruction complex. RNF43/ZNRF3 targets FZD to antagonise Wnt signalling. The accumulative result is a transcriptional “off” state. (**B**) Wnt binding causes dimerisation of FZD and LRP5/6. The receptor complex recruits the destruction complex to the cell membrane, and DVL assists the interactions between LRP5/6 and AXIN. Cytoplasmic β-catenin, thus, can be relieved from proteasome-dependent degradation. Stable β-catenin accumulates in the cytoplasm, followed by nuclear transport. There, β-catenin displaces repressive complexes and forms active complexes through associations with transcription factors and recruitment of coactivators and chromatin remodelers to upregulate Wnt responsive targets. RNF43/ZNRF3′s inhibition on the Wnt pathway can be counteracted by binding of RSPO to LGR5.

Wnt signaling is a growth control pathway that can regulate many biological processes ranging from development and evolution to adult homeostasis. Wnt signaling includes two branches, canonical (β-catenin-dependent activity) and non-canonical (β-catenin-independent activity) Wnt pathways. β-catenin is composed of a central region, including twelve imperfect Armadillo repeats, which are flanked by different domains in N- and C-terminus, respectively [1]. It acts as a crucial nuclear effector of the canonical Wnt pathway. WNT proteins are important intracellular ligands that stimulate the canonical Wnt pathway. In the absence of a WNT ligand, cytoplasmic β-catenin is regulated by the destruction complex, mainly containing Adenomatous polyposis coli (APC), Axis inhibition protein (AXIN), Glycogen synthase kinase 3 (GSK3) and Casein kinase 1 (CK1) for final degradation [2,3,4]. The destruction complex induces continuous elimination of β-catenin, thus impeding its nuclear transport, so that expression of Wnt target genes is switched off. WNT ligand binding to the FZD-LRP5/6 receptor complexes stabilises cytoplasmic β-catenin via either inhibition of phosphorylation of β-catenin combined with the disassembly of the destruction complex [5,6,7,8] or inactivation of ubiquitination and prosomal degradation of β-catenin in the intact destruction complex [9]. Once stabilised, cytoplasmic β-catenin accumulates and translocates to the nucleus where it binds to transcription factors, such as the TCF/LEF family [10], together with the recruitment of different nuclear regulators, to activate downstream transcriptional cascades of the Wnt pathway (Figure 1). Several mutations in the pathway can cause ligand-independent β-catenin stabilisation, which, thus, contributes to oncogenic β-catenin-regulated transcriptional activity [11].

**Figure 2:**
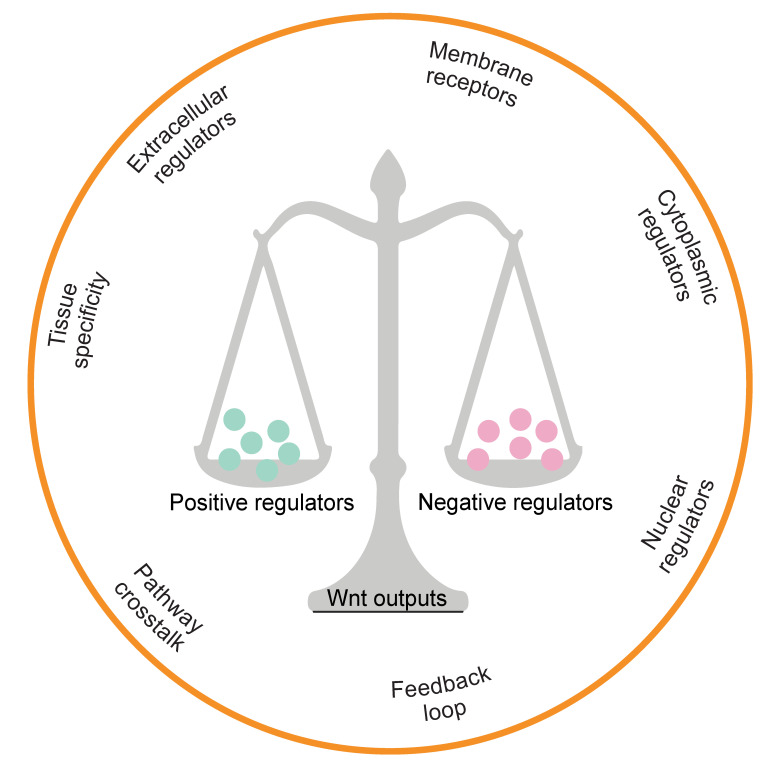
Multidimensional regulators in the Wnt signalling pathway. The hallmark determinants of β-catenin transcriptional output are depicted.

Moreover, many regulators have been identified which control β-catenin’s subcellular localisation, nuclear abundance or transcriptional activity (Table 1). Negative feedback loops, comprising AXIN or RNF43/ZNRF3, which are themselves Wnt downstream targets, exemplify the necessity of tight homeostatic control. The pathway is further complicated by the presence of another regulatory axis, LGR5-RSPO-RNF43/ZNRF3 [12,13,14]. Together, the involvement of multiple regulatory factors that can translate Wnt signals into different downstream readouts provides an explanation for the diversity and complexity of Wnt signalling (Figure 2).

Based on its fundamental functions in maintaining homeostasis, it is not surprising that inappropriate activation of the Wnt pathway contributes to many human diseases, including cancers [50]. Loss-of-function APC mutations are pathognomonic for colorectal cancer syndrome familial adenomatous polyposis (FAP) and contribute to the great majority of sporadic colorectal cancer development. Elegant work in both murine models of intestinal neoplasia and human cancer cell lines has highlighted the critical role for the oncoprotein MYC as an essential downstream driver of Wnt/β-catenin driven oncogenicity followed by APC loss [51]. More recently, it has been reported that Wnt signalling can further facilitate MYC accumulation by post-transcriptional mechanisms that lead to enriched availability of cytoplasmic pools of *MYC* transcripts [52]. Intriguingly, APC restoration can reverse the MYC-driven oncogenic state in colorectal cancer (CRC) and reestablish normal tissue homeostasis [53]. This demonstrates the crucial roles of APC mutations in both initiating and maintaining intestinal neoplasia and underscores the importance of the Wnt pathway as a potential therapeutic target for CRC.

The non-canonical Wnt pathway (β-catenin-independent activity) contains two well-recognised cascades, the planar cell polarity pathway (Wnt/PCP) and the Wnt calcium signalling pathway (Wnt/Ca^2+^). Some components, such as FZD and DVL, are shared by both the canonical and non-canonical Wnt pathways. It has been identified that canonical WNTs can be antagonised by non-canonical WNTs [54]. Deregulation of the canonical Wnt pathway has been implicated in cancers, but the roles of the non-canonical Wnt pathway in cancers remain largely unexplored. Some reports suggest the tumour-promoting [55,56] or repressive [57] functions of non-canonical Wnt pathway in CRC development. Voloshanenko et al. proposed that WNT5A/B (the non-canonical ligands) regulate the expression of non-canonical Wnt target genes, such as *PLOD2* and *HADH,* which are necessary for the proliferation and viability of CRC cells [56]. Recent work has also identified a direct role for WNT5A in promoting the migration and invasion of CRC cells [55]. On the other hand, WNT5A-driven phosphorylation of RORα, an orphan nuclear receptor, inhibits the expression of canonical Wnt target genes in CRC [57]. The complexities of the opposing forces from the canonical and non-canonical Wnt pathway are underscored by recent work showing the existence of coordinated canonical and non-canonical Wnt pathway signalling via simultaneous WNT3A activation and ROR2 inhibition, respectively, in colorectal cancer cells [58]. Collectively, further studies on the non-canonical Wnt pathway and its crosstalk with the canonical Wnt pathway will help improve our understanding of the multi-dimensional aspects of Wnt signalling in colorectal cancer.

Refined regulation of the Wnt pathway can activate or repress the expression of specific genes to exert various biological functions. The identification of positive and negative regulators would enable unravelling of the underlying regulatory mechanism of β-catenin-dependent transcription. However, despite intensive studies, we still have only begun to unravel the complexities of this transcriptional network. Herein, we focus on a review of nuclear regulators involved in the transcriptional activity of Wnt/β-catenin signalling with an emphasis on those associated with colorectal cancers. Depicting a network of regulators promoting physiological and/or oncogenic signal cascades can assist in identifying those representing vulnerabilities of specific cancer types, therefore directing the development of drug and treatment strategies.

## 2. TCF/LEF-Dependent β-Catenin Transcription Regulation

Wnt pathway-related gene expression is transduced by the nuclear effector β-catenin. Due to lacking a DNA-binding domain, β-catenin transcribes Wnt-responsive genes by way of interaction with TCF/LEF family members. Mosimann et al. proposed a simplified model to depict how the β-catenin-TCF/LEF complex sequentially recruits and interacts with activators to initiate and propagate transcription activity [59]. Most of the transcriptional activators bind to the C-terminus of β-catenin. However, some regulators bind to β-catenin N-terminus, such as BCL9/Pygo, which, thus, may be specific regulators of Wnt-induced transcription activity. In vertebrates, BCL9/9L interacts with Pygo to bind to the histone H3 tail methylated at lysine 4 (H3K4me), to promote Wnt-regulated transcription [60]. Moreover, a recent report indicates interactions between BCL9/9L, Pygo and nuclear co-receptor complexes. BCL9/9L acts as a constitutive component in the Wnt enhanceosome and rearranges under the Wnt stimulation, thus being considered as a regulator to integrate signals from hormones and Wnt [61]. In addition to its function as a scaffolding protein for Pygo, BCL9/9L has also been implicated in mediating transcriptional responses independent of Pygo in a cell type-specific manner. This is supported by a transcription activation domain found in the C-terminus of BCL9/9L, which, together with CREB-binding protein (CBP)/p300 and transformation/transcription domain-associated protein (TRRAP)/GCN5, facilitates β-catenin-mediated transcriptional activity [62]. In colon cancer, BCL9L appears to attenuate β-catenin’s association in adherens junctions, promoting its nuclear translocation to upregulate Wnt signalling [44]. In addition, BCL9 plays a β-catenin-independent role in promoting CRC via regulating the expression of stromal and neural associated genes [63]. Likewise, in addition to behaving as a co-activator of β-catenin, Pygo may remodel chromatin via a PHD domain to improve the accessibility of TCF/LEF or β-catenin to the transcription responses in CRC [64].

In addition to BCL9/Pygo, various nuclear mediators have been identified, and their existence helps explain, to some extent, how β-catenin is recruited to the promoters of Wnt target genes and how it complexes with TCF/LEF family members. For example, Transducin β-like protein 1 (TBL1) and TBL1-related protein (TBLR1) have been identified as a cofactor exchange complex for transcriptional activation [65]. Wnt-induced interaction between TBL1–TBLR1 and β-catenin can mutually promote their migration to the promoters of Wnt targets following by removing repressors, TLE1 and histone deacetylase 1 (HDAC1), to stimulate Wnt target gene expression [66]. Furthermore, PCNA-associated factor (PAF) not only directs β-catenin to the promoters of Wnt target genes but also works as a molecular adaptor responsible for the recruitment of EZH2. A non-canonical role for EZH2 has been identified in stimulating Wnt/β-catenin transcriptional output. In this regard, EZH2, independent of its methyltransferase function, plays a scaffolding role in bridging β-catenin to the Mediator complex and RNA polymerase II (RNAPII) [67].

**Figure 3:**
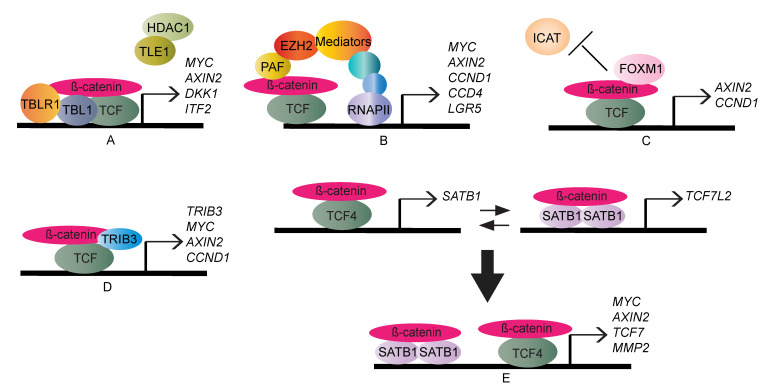
Schematic depicting the molecular mechanism of co-activators involved in TCF/LEF-dependent β-catenin transcriptional activities and their functional consequences. (**A**) TBL1 (Transducin β-like protein 1) and TBLR1 (TBL1-related protein) interact with nuclear β-catenin, and the interaction promotes their binding to the promoters of Wnt target genes. Subsequently, TLE1 and histone deacetylase 1 (HDAC1), two transcriptional repressors, are displaced to initiate downstream transcriptional cascades. (**B**) PAF (PCNA-associated factor) recruits EZH2, a histone-lysine N-methyltransferase, to the β-catenin-TCF/LEF complex. Consequently, EZH2 and the Mediator complex recruit RNA polymerase II-dependent transcriptional machinery to activate Wnt target gene expression. (**C**) Under Wnt stimulation, stabilised FOXM1 (Forkhead box protein M1) can release β-catenin from ICAT (aka CTNNBIP1, β-catenin-interacting protein 1), thus abolishing its inhibition on β-catenin-TCF/LEF complex to transcribe Wnt target genes. (**D**) TRIB3 (Tribbles pseudo-kinase 3) participates in a positive feedback loop. TRIB3, a downstream target of the Wnt pathway, can stabilise the β-catenin-TCF4 complex, thus improving its transcriptional activity. (**E**) SATB1 (Special AT-rich sequence-binding protein-1) regulates TCF4 expression, and complexes with β-catenin to transcribe Wnt downstream genes.

Some regulators are capable of promoting the assembly of the β-catenin-TCF/LEF complex to enhance the transcriptional activity of the Wnt pathway. For example, a cell cycle regulator, Forkhead box protein M1 (FOXM1), stabilised upon Wnt activation, antagonises β-catenin-interacting protein 1 (ICAT), an inhibitor for β-catenin-TCF/LEF complex. In this manner, FOXM1 enables the localisation of β-catenin to promoter elements and facilitates the formation of the transcriptional complex [68]. Similarly, PLD1 (Phospholipase D1), a downstream target of Wnt signalling, enhances the interaction between β-catenin and TCF4 by downregulating the expression level of ICAT, thus potentiating β-catenin-dependent transcriptional activity [69,70]. Moreover, some regulators induce a positive feedback loop to enhance Wnt/β-catenin outputs. Tribbles pseudo-kinase 3 (TRIB3), a stress sensor, has been suggested as a marker of CRC patients [71]. In the Wnt pathway, activated β-catenin transcriptional complex improves *TRIB3* expression. TRIB3 brings β-catenin in close proximity to TCF4 to transactivate Wnt targets [72]. Special AT-rich sequence-binding protein-1 (SATB1), a chromatin organiser, participates in a reciprocal and bidirectional transcriptional regulation. Β-catenin-TCF4 induces the expression of SATB1 and β-catenin-SATB1, in turn, maintains the transcription of *TCF7L2* (encoding TCF4). It is assumed that the promoters of Wnt-regulated genes could be occupied by these two complexes to modulate their transcriptional outputs [73] (Figure 3).

**Figure 4:**
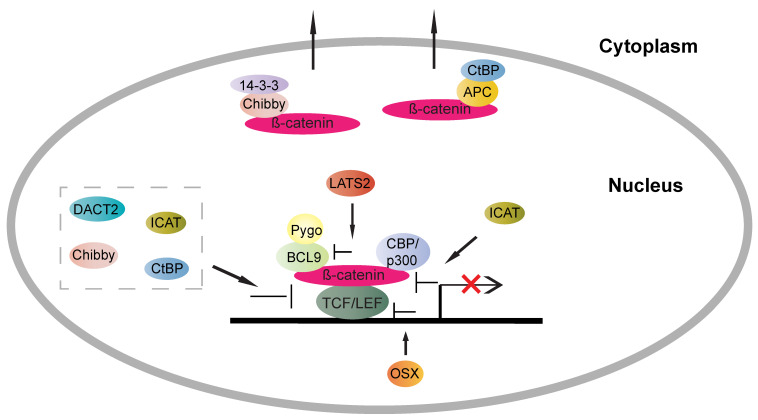
Schematic representing the molecular mechanism of transcriptional repression in TCF/LEF-dependent Wnt outcomes. Β-catenin-TCF/LEF-regulated transcription can be inhibited via various mechanisms, including 1. interfering with the interaction between β-catenin and TCF/LEF, as shown for ICAT (aka CNNTBIP1, β-catenin-interacting protein 1), DACT2 (Dapper homolog 2), Chibby, CtBP (C-terminal-binding protein 1), 2. binding with β-catenin to facilitate its nuclear export, such as Chibby and CtBP, 3. interfering with the interaction between β-catenin and its co-activators, such as ICAT—targeting the β-catenin-p300 complex and LATS2—targeting the β-catenin-BCL9 complex and 4. interfering with the binding of TCF/LEF to DNA, such as OSX (Osterix).

In *Drosophila*, binding of Armadillo (β-catenin) to TCF/LEF or binding of β-catenin-TCF/LEF complex to DNA leads to repressed expression of some genes, such as *Cdh1* (encoding E-cadherin) [74] and *Sr* (encoding Stripe) [75]. This effect has been linked to the utilisation of non-common TCF/LEF consensus binding sites [76]. Additionally, a switch for β-catenin-TCF/LEF complexes from an active to repressive state can be achieved by disrupting the interaction between β-catenin and TCF/LEF. Nuclear ICAT can impede the binding of β-catenin with TCF/LEF and p300 (an agonist of the Wnt pathway) simultaneously to inhibit Wnt signalling [77,78]. Whereas, cytoplasmic ICAT can interfere with β-catenin-mediated own degradation in the destruction complex in the context of activated Wnt or the APC truncated state [79]. Dapper homolog 2 (DACT2) inhibits the Wnt pathway through two separate mechanisms. Nuclear DACT2 disrupts the interaction between β-catenin and LEF1, and cytoplasmic DACT2 represses β-catenin nuclear transport via restoring E-cadherin–β-catenin complex [80]. Chibby, a newly identified β-catenin antagonist, can disrupt the association between β-catenin and LEF1, and promotes β-catenin nuclear export via a chibby-14-3-3-β-catenin complex [81,82]. C-terminal binding protein, CtBP, has been implicated in both the positive and negative regulation of nuclear β-catenin activity. The inhibitory mechanisms of CtBP on transcriptional events are controversial, possibly via either assembling with TCF/LEF directly [83,84] or reducing β-catenin nuclear availability with the help of nuclear APC [85,86]. However, it is worth noting that CtBP has been demonstrated to also play a role as a transcriptional coactivator for specific Wnt/β-catenin target genes in a TCF dependent manner [87,88]. The ability of CtBP to function as a transcriptional repressor or activator depends on its conformational state. This is evidenced by the findings that CtBP dimers are implicated in transcription suppression, whereas CtBP monomers can induce the expression of Wnt/β-catenin targets [88]. In addition, LATS2 is a key component of the Hippo pathway, where it causes the retention and degradation of cytoplasmic YAP, thus inhibiting cell proliferation and tumourigenesis [89]. In Wnt signalling, LATS2, independent of its kinase ability, can target the interaction between β-catenin and BCL9 to inactivate downstream transcription activity [90]. Osterix (OSX), a zinc-finger containing transcription factor of the specificity protein (Sp) family, can lead to compromised β-catenin-TCF/LEF transcription regulation by both enhancing DKK1 expression level and impairing the binding of TCF/LEF to DNA [91] (Figure 4).

Identification of diverse nuclear regulators further supports that the activation of dysregulated Wnt pathway in cancer extends beyond genetic mutations, such as those involved in *APC* or *CTNNB1*. It is clear that future studies will be required to elucidate the mechanistic connection between these regulators. This will help broaden potential therapeutic targets and will help provide novel therapeutic methods for the cancers mainly driven by β-catenin-TCF/LEF complex, where APC mutation is uncommon.

## 3. TCF/LEF-Independent β-Catenin Transcription Regulation

TCF/LEF family members are postulated to be the final effectors of the β-catenin-dependent Wnt pathway [10]. However, accumulating evidence indicates β-catenin can interact with other transcription factors to mediate gene expression changes. This seems to provide one explanation for the surprising observations that TCF4 functions as a tumour suppressor in colon tumourigenesis [92,93]. β-catenin-driven malignant transformation can be mediated via its interaction with at least two distinct downstream effectors TCF4 and YAP1-TBX5. Tyrosine kinase YES1 can phosphorylate YAP1, thus directing β-catenin-YAP1-TBX5 to the promoters of pro-survival genes, such as *BCL2L1* and *BIRC5* [94]. On the other hand, nuclear YAP1 inhibits transcription in β-catenin-active colon cancers via regulating chromatin state through the SWI/SNF complex [95]. Nuclear activation of endogenous YAP/TAZ stabilises TLE, an antagonist of the β-catenin-TCF4 transcriptional complex, which causes Wnt signalling suppression and loss of intestinal stem cells [96]. Cytoplasmic YAP plays suppressive functions in Wnt signalling outputs. It can suppress Wnt signalling through diverse mechanisms, including 1. suppressing nuclear transmission of DVL, an agonist of Wnt pathway [97], and β-catenin itself [98] and 2. recruiting β-TrCP to inactivate β-catenin [99]. Additionally, TEAD4, a transcription factor of the Hippo pathway, can complex with TCF4 to potentiate the expression of a set of Wnt genes [100]. Altogether, nuclear YAP1 and TEAD4 can compete or assist with TCF4 to regulate Wnt target gene expression, thus hinting at TCF/LEF is not the only transcription factor to transduce Wnt signalling.

Functional interplay with accessory transcription factors provides the ability to fine-tune β-catenin-regulated gene expression. For example, the interaction between β-catenin and OCT4, a master regulator of stem cell pluripotency, promotes stem cell pluripotency via enhancing OCT4 transcriptional activity [101] and inducing β-catenin proteasomal degradation [102]. Some nuclear regulators interact with β-catenin to initiate specific transcription programs that enable cancer cells to adapt to hypoxic conditions in tumour growth [103]. For example, FOXO is a subfamily of transcription factors regulating apoptosis, proliferation and oxidative stress. FOXO can disrupt the β-catenin-TCF/LEF complex to suppress Wnt signalling, but can also assemble with β-catenin to promote the transcription of FOXO-regulated target genes [104,105]. In colorectal cancer cells, HIF-1α, the oxygen-sensitive subunit of hypoxia-inducible factor-1 (HIF-1), directly binds to β-catenin, thus impeding the interaction between β-catenin and TCF4. This inhibits the Wnt pathway but potentiates the expression of HIF-1α-dependent targets [106].

β-catenin can bind with other transcription factors, usually counteracting TCF/LEF-dependent transcription, to facilitate the expression of different sets of genes associated with various functions, such as pluripotency, pro-survival and tumour metabolism. Further supporting this view, Doumpas et al. indicated that β-catenin can swap between different transcription factors to regulate the expression of β-catenin-dependent genes when TCF/LEF are absent or the interaction between β-catenin and TCF/LEF is inhibited [107]. In sum, β-catenin regulates different stages of homeostatic and tumourigenic development through context-specific interactions with different accessory transcription factors. It is necessary that future work will be needed to identify the specific key regulators which recruit β-catenin to specific transcription factors and the downstream functional consequences.

SOX9 (sex-determining region Y (SRY)-box 9 protein), an intestinal crypt transcription factor, exerts crucial roles in regulating Paneth cell differentiation in the intestinal epithelium [108,109]. SOX9 is itself a Wnt downstream target gene, and its expression can inhibit intestinal lineage and differentiation markers such as *CDX2* and *MUC2* genes. This, in turn, helps to maintain a progenitor cell phenotype [110]. However, the roles of SOX9 in intestinal tumourigenesis remain controversial. According to some models, loss of SOX9 activity has been proposed to promote tumourigenesis. In one example, it has been postulated that SOX9 represses the expression of one of the claudin family polarity maintenance genes, *CLDN7* (encoding Claudin-7). Consistent with this, Claudin-7 overexpression was found to promote loss of tumour cell polarity and tumourigenesis in a SOX9 dependent manner [111]. Nevertheless, SOX9 has also been found to be overexpressed in some CRCs [112], and oncogenic functions have been reported for SOX9 in the context of colon tumour development [113]. Subsequent studies have alluded to a context-dependent role for SOX9 [114], which may provide an explanation for these contradictory results.

Additionally, it has been demonstrated that some transcription factors can antagonise the binding of the β-catenin-TCF/LEF complex at relevant genomic loci to differentially regulate gene expression. GATA6, belonging to the GATA family of zinc-finger transcription factors, is one such example. GATA6 competes with the β-catenin-TCF4 complex to bind to the enhancer elements of *BMP4* to inhibit its expression, ultimately restricting BMP signalling to differentiate tumour cells in CRC [115]. Furthermore, it can directly potentiate the expression of Wnt/β-catenin target gene *LGR5* in CRC cells [116]. These observations suggest that GATA6 cooperates with β-catenin at several levels to synergise Wnt and BMP signalling within the context of cancer initiation.

## 4. Mediator Kinase of β-Catenin-Mediated Transcriptional Output

The Mediator complex acts as a crucial component in transcription regulation, in light of its ability to transduce signals from pathway-specific transcription factors (TFs) to regulate RNA polymerase II (RNAPII) activity [117,118]. Depending on its functional configuration, the Mediator complex consists of 25 to 30 proteins, which can be classified into four submodules—head, middle, tail and Mediator kinase (Cyclin-dependent kinase 8 (CDK8), 19) module. The CDK8 module contains CDK8 (or its CDK19 paralog), Cyclin C (encoded by CCNC), MED12 and MED13 subunits. CDK19, a highly conserved paralog of CDK8 in vertebrates, shares conservation in the kinase and cyclin binding domain but is divergent at the C-terminal tail [119]. CDK8 and CDK19 are incorporated into the Mediator kinase module by way of binding MED12 in a mutually exclusive manner. Cyclin-dependent kinase (CDK) proteins mainly consist of two types of members, cell cycle progression CDKs, such as CDK1, CDK2 and CDK4/6, and transcriptional CDKs, such as CDK7, CDK9 and CDK8/19 [120]. These transcriptional CDKs complex with their cyclin subunit to phosphorylate the C-terminal domain (CTD) of RNAPII, regulating transcription initiation, elongation and mRNA processing [121]. Unlike CDK7 and CDK9, CDK8/19 activity is dispensable for general transcription regulation and normal cell growth. Recently, the CDK8 kinase function has been linked to an “activation helix” placed by MED12 close to the CDK8 T-loop [122]. CDK8/19 acts downstream of several transcription factors, such as HIF-1α [123] and NFκB [124], to transcribe their target genes. In addition, apart from its action on RNAPII, CDK8 phosphorylates some TFs, such as SMAD, to regulate β/BMP signalling [125]. CDK8/19 is associated with diverse TFs to selectively enhance the expression of silent genes, implying their regulation is in a context-specific manner.

CDK8/19 has been implicated in several cancers, especially those induced by dysregulated gene expression rather than mutations [126]. It has been more than a decade since CDK8 was identified as an oncoprotein in colon cancer. CDK8 was found to act, in a kinase-dependent manner, as a driver of β-catenin-dependent transcription and colorectal cancer proliferation [127]. The underlying molecular mechanisms of how CDK8 regulates β-catenin-dependent transcription are less-well defined, especially considering that the critical kinase substrates of CDK8 that mediate this activity are yet to be identified. Converging genetic studies have demonstrated that the ability of CDK8 to drive β-catenin-regulated gene expression is likely Mediator complex dependent. Aside from CDK8, Mediator kinase submodule proteins MED12 and MED13 have been implicated in recruiting Mediator to Wnt-directed target genes and regulating β-catenin transcriptional output [128,129]. Furthermore, repressing the CDK8 co-factor, Cyclin C, produces similar influences on β-catenin-mediated transcription as the inhibition of CDK8 [127]. Nevertheless, it has also been proposed that CDK8 can exert its effects on β-catenin indirectly via potentially Mediator-independent mechanisms. Morris et al. suggested pRB and CDK8 can counteract the inhibition of E2F1 on β-catenin-regulated transcription to enhance the expression of Wnt/β-catenin genes [130]. Indeed, chromosomal copy number gains in both *CDK8* and *RB1* loci on 13q in colorectal cancer cells provide a clear explanation of how they select optimal conditions to antagonise E2F1 suppressions [127,130]. E2F1 can post-translationally degrade β-catenin [130] and can activate ICAT which disrupts β-catenin-TCF/LEF complex [131]. The physical interaction between CDK8 and E2F1 at the promoters of β-catenin targets, such as *MYC,* provides a basis for understanding how Mediator kinase may enhance β-catenin transcriptional output [130]. Subsequent studies have further demonstrated that CDK8 phosphorylates Ser375 on E2F1 in colon cancer cells. This phosphorylation alleviates the E2F1 repressive effects on β-catenin-associated transcription [132]. Together, these data imply that E2F1 could be one of the crucial substrates of CDK8 to enable its regulation on Wnt/β-catenin transcriptional output. Moreover, a recent study identifies YAP as a novel substrate of CDK8. In Hippo signalling, YAP is phosphorylated directly by CDK8 to induce its function, and the phosphorylated YAP can be regulated further by Zyxin, a zinc-binding phosphoprotein, to promote cell proliferation and migration in CRC [133]. In conclusion, CDK8 kinase activity plays an essential role in driving oncogenic events. It can activate different substrates that are associated with intestinal neoplasia and tumour progression. Accordingly, it is quite possible that therapeutic interventions targeting its kinase activity could have clinical value in the treatment of CRC.

## 5. Chromatin States Regulate β-Catenin-Mediated Transcriptional Output

**Figure 5:**
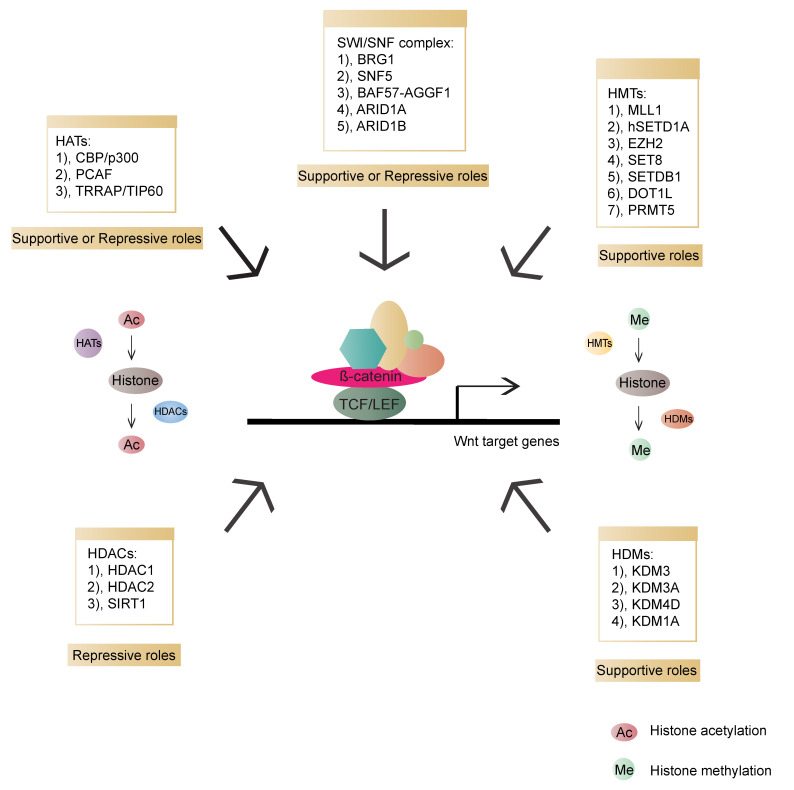
Overview of chromatin modifiers involved in β-catenin-dependent transcriptional regulation and their roles in the Wnt pathway. HATs (histone acetyltransferases, transferring acetyl groups to histones): CBP (CREB-binding protein)/p300, PCAF (p300/CBP-associated factor), TRRAP (Transformation/transcription domain-associated protein)/TIP60; HDACs (Histone deacetylase, removing acetyl groups from histones): HDAC1 (Histone deacetylase 1), HDAC2 (Histone deacetylase 2), SIRT1 (NAD-dependent deacetylase sirtuin-1); HMTs (Histone methyltransferases, transferring methyl groups to histones): MLL1 (Mixed-lineage leukemia 1 or Histone-lysine N-methyltransferase 2A), hSETD1A (human SET domain containing protein 1A), EZH2 (Enhancer of zeste 2 polycomb repressive complex 2 subunit), SET8 (SET domain-containing protein 8), SETDB1 (SET domain bifurcated 1), DOT1L (DOT1 like histone lysine methyltransferase), PRMT5 (Protein arginine N-methyltransferase 5); HDMs (Histone demethylases, removing methyl groups from histones): KDM3 (Lysine-specific demethylase 3), KDM3A (Lysine demethylase 3A), KDM4D (Lysine-specific demethylase 4D), KDM1A (Lysine demethylase 1A); SWI/SNF: BRG1 (Transcription activator BRG1), SNF5 (BRG1-associated factor 47), BAF57 (BRG1-associated factor 57), AGGF1 (Angiogenic factor with G patch and FHA domains 1), ARID1A (AT-rich interactive domain-containing protein 1A), ARID1B (AT-rich interactive domain-containing protein 1B).

Chromatin remodeling is an essential step for transcription regulation. The nucleosome, the basic subunit of chromatin, is composed of histones H2A, H2B, H3, H4 and 146 base pairs (bp) of DNA. These histone tails undergo diverse post-translational modifications, such as acetylation, methylation, phosphorylation, ubiquitination, sumoylation and ADP ribosylation. Such modifications can increase or decrease the accessibility of DNA for transcriptional regulators to coordinate gene expression [134,135,136]. Histone acetyltransferases (HATs) and deacetylases (HDACs) are associated with enhanced and decreased acetylation, which mark gene activation and repression, respectively [137]. Furthermore, histone methylation can both alter chromatin accessibility and epigenetically activate or inactivate gene expression. The dynamic balance between histone methylation and demethylation is mediated by histone methyltransferases (HMTs) and demethylases (HDMs), respectively [138]. In addition to histone modification, chromatin remodeling is also an important epigenetic event. ATP-dependent remodeling complexes can alter the location and configuration of the nucleosome, and the changed chromatin can participate in either gene activation or inhibition [137]. Epigenetic regulation is involved in various events, including transcription, replication, repair and genome stability. Lastly, chromatin modifiers have been reported to have essential influences on cancer etiology [139,140,141]. In Wnt signalling, various chromatin modifiers have been identified as activators or repressors of Wnt/β-catenin transcriptional outputs (Figure 5).

### 5.1. Histone Acetyltransferases (HATs)

HATs can transfer acetyl groups to lysine tails on histones to improve/inhibit gene expression [142]. CBP and its paralogue p300 are members of the mammalian HATs. They may function as scaffolds to associate simultaneously with the basal transcription factors such as TBP, TFIIB and/or RNA polymerase II and with upstream transcription factors to form stable transcription complexes [143]. Additionally, CBP/p300 can acetylate histones and non-histone transcription-associated proteins to work as HATs and factor acetyltransferases (FATs), respectively [144]. CBP and p300 have been found to regulate Wnt/β-catenin target gene expression. In *Drosophila*, dCBP can acetylate dTCF to decrease its affinity with Armadillo (β-catenin) to inhibit Wingless signalling [145]. Conversely, dCBP can also associate directly with Armadillo (β-catenin), upon which it is directed to a Wnt responsive element to activate Wg targets [146]. In vertebrates, CBP and p300 are reported to positively promote the expression of Wnt target genes either via interacting with β-catenin [147,148,149,150] or via acetylating β-catenin itself. Despite high homology between CBP and p300, converging evidence suggests that their functions are not completely redundant, which is supported by observations that CBP and p300 acetylate different sites on β-catenin as well as mediate the transcription of different Wnt/β-catenin target genes [151,152,153]. Indeed, acetylation of the β-catenin protein has been pinpointed to Lys49 and Lys345 by CBP [154] and p300 [155], respectively. The utilisation of these different HATs to regulate β-catenin activity may be context-specific. For example, dynamic switching from CBP to p300 has been linked with adult progenitor cell differentiation [156]. In addition to CBP/p300, acetyltransferase PCAF has also been demonstrated to promote β-catenin activity in an acetylation dependent manner. PCAF was shown to directly acetylate β-catenin, thereby inhibiting its degradation and promoting its nuclear accumulation and activity [157]. Lastly, the TRRAP/TIP60 histone acetyltransferase complex participates in β-catenin-regulated transcription. TIP49a/b (also known as Pontin52/Reptin52) and TIP60 are subunits of the complex [158]. TIP49a and TIP49b all bind directly with β-catenin and TBP, whereas they regulate β-catenin-dependent transcription in an antagonistic manner. TIP49a acts as an important regulator for β-catenin-activated transcription in normal and neoplastic cells, while TIP49b inactivates gene expression mediated by the β-catenin and TCF/LEF complex [159,160]. For example, the expression of *KAI1*, a metastasis suppressor gene, is triggered by the P50-Pontin-TIP60 complex but is repressed by the P50-β-catenin-Reptin complex [161]. In general, these data illustrate the general principle that HATs facilitate Wnt/β-catenin mediated transcriptional output and are critical modulators of β-catenin functions at the level of intestinal homeostasis and tumour pathogenesis.

### 5.2. Histone Deacetylases (HDACs)

HDACs can remove acetyl groups from lysine tails on histones to counteract the activity of HATs. This causes chromatin to be compacted, thus inactivating the transcription of target genes. HDAC1 can combine with LEF1 to suppress Wnt/β-catenin target gene expression, whereas β-catenin can displace HDAC1, followed by interaction with LEF1 to render the new complex ready for activation [162]. On the other hand, HDAC1/2 can compete with β-catenin to interact with TCF4 to exert their repression on Wnt targets [163]. NAD-dependent deacetylase sirtuin-1 (SIRT1) inhibits β-catenin transcriptional activity and drives the cytoplasmic transfer of oncogenic β-catenin, which represses colon tumour formation [34]. Together, HDACs are generally responsible for transcription repression. Rather than silencing tumour suppressor genes, HDACs exert suppressive roles in Wnt signalling, which helps maintain the balance of intestinal homeostasis.

### 5.3. Histone Methyltransferases (HMTs)

Histone methylation includes histone lysine methylation and histone arginine methylation. Histone lysine methylation can happen on lysine residues, such as Lys4, Lys9, Lys27, Lys36 and Lys79, of histone H3 and Lys20 of histone H4 via mono-, di-, or trimethylated manners [164]. In addition, a SET domain is contained in almost all lysine histone methyltransferases (HMTases), and it imparts the catalytic activity necessary for methylating lysine residues on histones H3 or H4 [165].

Lysine methyltransferases family 2 (KMT2) can methylate Lys 4 of histone H3 (H3K4), which is related to gene activation in eukaryotes [166]. The family exists in the COMPASS complex and consists of five members—SET1, mixed-lineage leukemia 1 (MLL1), MLL2, MLL3 and MLL4 in mammalian cells [167]. MLL1 (encoded by *KMT2A*), the member of a mammalian trithorax-group (trx-G) gene, plays an essential role in maintaining the expression of the Hox gene during development [168]. Nascent MLL1, via a cleavage modification catalyzed by Taspase1, can be re-assembled into mature MLL1 complex. In human acute leukemia, inter-chromosomal translocations involve in the *KMT2A* gene, leading to the formation of new chimeric MLL1 fusion proteins, which lose the H3K4 methyltransferase domain [169,170,171,172]. Additionally, MLL1 has been linked with CRC development [173]. MLL1 is involved in regulating Wnt/β-catenin target gene expression [86]. Subsequent studies have shown that β-catenin can configure chromatin via MLL1-dependent H3K4 trimethylation (H3K4me) in the salivary gland and head and neck tumours. MLL1 indirectly binds with β-catenin via CBP, and the complex usually works with TCF4 to transcribe Wnt/β-catenin target genes [174,175]. In addition, in brain glioma, Pygo2 has been reported to promote the recruitment of MLL1 and MLL2 complex to upregulate H3K4me3 level and Wnt target gene transcription [176]. Human SET domain containing protein 1A (hSETD1A), another member of the trithorax (TrxG) family, can interact with β-catenin so as to be recruited to the promoters of Wnt-associated genes. After that, it can methylate H3K4 to assemble transcription preinitiation complex and then activate transcription [177].

EZH2, a catalytic subunit of the polycomb repressive complex 2 (PRC2), targets H3K27 methylation to inactivate gene transcription [178]. In hepatocarcinogenesis and osteoarthritis, EZH2 can activate Wnt/β-catenin signalling by enhancing H3K27me3 on *SFRP1* promoter, a Wnt inhibitory gene [179,180]. Additionally, SET8 (encoded by *KMT5A*) is a member of the SET domain-containing methyltransferase family and is specifically responsible for H4K20 monomethylation to control gene expression [181]. In Wnt/β-catenin signalling, SET8 has been shown to directly associate with TCF4, likely through H4K20 monomethylation regulation at the promoters of Wnt-associated gene, to promote their expression in mammalian cells. Of note, it only performs this function upon β-catenin displacement of the transcriptional co-repressor Groucho/TLE from TCF4 upon Wnt activation [182]. Furthermore, SET8 has been suggested to enhance β-catenin-regulated transcription by promoting its dissociation from adherens junctions [183]. SET domain bifurcated 1, (SETDB1), is an H3K9 histone methyltransferase. SETDB1 can be recruited by activated Notch signalling to the promoters of Wnt-response genes, thus inhibiting their expression in CRCs [184].

Unlike other lysine HMTases, DOT1L functions as H3K79 methyltransferase despite lacking a SET domain [185]. In leukemia, DOT1L can specifically bind to MLL1 fusion proteins to promote tumourigenesis [172]. In addition, DOT1L-mediated H3K79 methylation is essential for maintaining intestinal homeostasis. AF10 and DOT1L can combine with the β-catenin-TCF4 complex to induce H3K79 methylation, directing transcription elongation at Wnt target genes [186]. Successful isolation of a DOT1L-containing multi-subunit complex suggests physical associations between DOT1L and β-catenin. Moreover, H2B monoubiquitination seems to be a prerequisite for DOT1L-induced H3K79 trimethylation to mediate Wnt gene expression [187]. On the other hand, in the context of cartilage homeostasis, DOT1L inactivates Wnt signalling in a SIRT1 dependent manner [188].

Additionally, histone arginine residues can also be methylated by protein arginine methyltransferases (PRMTs). PRMTs have nine isoforms in mammalian genomes and catalyze three different types of arginine methylation, including monomethylation as well as asymmetric and symmetric dimethylation. PRMT5 is responsible for symmetric demethylation, which modifies histones H3 and H4 to inhibit gene transcription. In lung and blood cancers, PRMT5 is overexpressed and works as a transcriptional repressor to inhibit tumour suppressor expression [189,190]. In lymphoma, PRMT5 promotes Wnt/β-catenin signalling through directly inhibiting the expression of *AXIN2* and *WIF1*, two antagonists of the pathway, and indirectly activating AKT/GSK3β signalling [191]. However, in chronic myelogenous leukemia, PRMT5 is suggested to promote DVL3 expression epigenetically, an agonist of Wnt signalling, to activate the pathway [192]. Moreover, in hepatocellular carcinoma (HCC), PRMT5 and β-catenin may competitively interact with metadherin (*MTDH*), an oncoprotein, which regulates the Wnt pathway to promote HCC metastasis. *MTDH* overexpression causes the transport of PRMT5 from the nucleus to the cytoplasm, followed by that of β-catenin from the cytoplasm to the nucleus, which upregulates Wnt signalling [193].

Overall, HMTs play critical roles in promoting Wnt signalling by promoting the expression of agonists or inhibiting the expression of antagonists of the pathway. Moreover, dysregulation of HMT activity in the context of the Wnt pathway has been linked to many tumour types, but the prevalence of different enzymes is identified in specific organs, suggesting their tissue-specific tumour-promoting functions.

### 5.4. Histone Demethylases (HDMs)

Protein lysine demethylases (KDMs) can modify chromatin by demethylation of histone or non-histone protein lysine residues. Recently, some KDMs have been implicated in human CRC mainly through epigenetic modifications on H3K9, which augments Wnt/β-catenin signalling. H3K9 is involved in gene silencing, and KDM3 can directly remove suppressive H3K9me2 marks to activate Wnt downstream gene expression epigenetically. KDM3 can also drive MLL1-dependent H3K4 methylation to promote the binding of BCL9 and Pygo2 to chromatin, thus contributing to Wnt/β-catenin related transcription activation [194]. Additionally, KDM3A (aka JMJD1A) can also erase repressive marks from H3K9me2, in conjunction with β-catenin, to enhance transactivation of Wnt/β-catenin target genes [195]. Another lysine demethylase, KDM4D (aka JMJD2D), can associate physically with β-catenin and remove methyl modifications from H3K9me3 to promote β-catenin-regulated gene expression [196]. While the current literature suggests that KDMs remove repressive epigenetic modifications on Wnt/β-catenin targets to promote colon tumourigenesis, the context and temporal nature of these effects require further investigation. In addition, KDM1A (aka LSD1), independent of its demethylase catalytic activity, promotes intestinal tumourigenesis by upregulating Wnt signalling via inhibiting the expression of *DKK1*, an antagonist of the Wnt pathway [197]. These data highlight the emerging role of histone demethylases in directly modulating β-catenin transcriptional output.

### 5.5. SWI/SNF Complex

SWI/SNF is a conserved subfamily of ATP-dependent chromatin-remodeling complexes from yeast to human [198]. They can utilise ATP to modify chromatin and shuffle nucleosomes so as to act as an activator or repressor of gene expression. SWI/SNF complexes may include, in a mutually exclusive manner, either the BRM or BRG1 ATPase subunit, which is essential to drive chromatin remodeling [199]. In addition, the SWI/SNF complex performs its functions via engaging with specific transcription factors due to a lack of intrinsic DNA-binding specificity within the complex itself [199,200]. For example, the SWI/SNF complex can be recruited to the promoters of Wnt targets via interaction between β-catenin and BRG1, which regulates transcriptional activity in the context of Wnt/β-catenin signalling [201]. Moreover, loss of BRG1 reduces RNF43 expression, a suppressor of Wnt/β-catenin signalling, thus promoting Wnt/β-catenin pathway activation and CRC metastasis [202]. Remarkably, telomerase and Wnt/β-catenin pathway can also converge, via the association between TERT and BRG1, to synergistically mediate stem cell development [203]. Another subunit, SNF5, exerts suppressive effects on the Wnt/β-catenin pathway. Its loss leads to hyperactivation of the signalling owing to the uncoupling of Wnt target activation at a chromatin level from the control of the upstream Wnt pathway [204]. Additionally, angiogenic factor with G patch and FHA domains 1 (AGGF1), a secreted angiogenic growth factor, can interact with BRG1-associated factor 57 (BAF57), a subunit of SWI/SNF complex, and β-catenin to promote the expression of LEF1 and AXIN2, two Wnt/β-catenin target genes [205].

The BAF chromatin-remodeling complex, an analogue of the SWI/SWF complex, has also recently been reported to regulate Wnt signalling. For example, AT-rich interactive domain-containing protein 1 (ARID1), the largest subunit of the complex, possesses two isoforms—ARID1A and ARID1B. The deficiency of ARID1A can drive CRC without APC mutations owing to the dysregulation of enhancer activity [206]. Subsequently, it has been reported that ARID1A is indispensable for intestinal homeostasis maintenance via regulating the Wnt pathway and SOX9 expression [207]. Lastly, in the context of neurodevelopment, ARID1B interacts with β-catenin via BRG1 to suppress Wnt-mediated downstream transcription. Mutations in ARID1B partly or completely abrogate its binding sites with BRG1, thus alleviating its suppressive effects on the Wnt/β-catenin pathway [208].

## 6. Therapeutic Strategy

The clinical implications of targeting the Wnt signalling pathway has been recognised for decades. Nevertheless, this has not been translated into the clinic for reasons detailed below. Dysregulated Wnt pathway can be targeted at three different levels: the Wnt-receptor complex, the cytoplasmic destruction complex and the nuclear β-catenin-TCF/LEF complex. Regulators involved in downstream transcriptional levels emerge as the most attractive targets for drug development. The implications of APC loss in the vast majority of CRC make inhibitors targeting Wnt signalling components upstream of the destruction complex ineffective. One exception is CRCs harbouring RSPO2/3 fusions [209]. While accounting for only 1–3% of CRC cases, the ability to therapeutically target RSPO2/3 fusion driven CRCs offers a proof of concept evidence that specific cancer targeting for the Wnt pathway can be achieved [210]. Recent studies in human colon organoids and in in silico genetic interaction analysis provide a model of how homeostatic and oncogenic Wnt responses can be untangled and stratified [211,212]. Diverse transcriptional regulators provide potential avenues to identify those only contributing to tumourigenesis. This diversity within this growing toolbox of targets offers opportunities to design rational therapies that will specifically target oncogenic signalling with minimal normal toxicities.

Disrupting protein–protein interactions in transcriptional complexes opens a new therapeutic window for anti-cancer drug development, although the transient nature of some interactions hinders effective drug development. Currently, most of the inhibitors targeting transcriptional level in Wnt pathway attempt to disrupt β-catenin-TCF4 interactions [213,214,215,216,217,218]. In addition, inhibitors interfering with the interaction between β-catenin and CBP may be suggested as attractive candidates [219,220,221,222]. Recently, in APC-deficient models, it was demonstrated that abrogation of BCL9/9L cannot affect intestinal homeostasis, but can negatively influence intestinal neoplasia, thus highlighting the therapeutic potential of targeting the interaction between β-catenin and BCL9/9L [223,224]. Kinase inhibitors are a mainstay of cancer therapies. The ability of a specific CDK8/19 kinase inhibitor, CCT251545, inhibits Wnt pathway-induced tumours in mouse models [225]. Similarly, another kinase, YES1, implicated in YAP dependent β-catenin transcriptional output, can be inhibited by CH6953755 to restrict YES1-YAP1-dependent tumour growth [94,226]. Lastly, NSC48300, a pharmacologic inhibitor for Taspase1, can effectively inactivate breast and brain tumour growth via preventing the formation of a mature MLL1 complex [227]. Current inhibitors and their drug development status are summarised in Table 2.

Epigenetic modifications are reversible, thus attracting extensive attention to targeting abnormal epigenetic events so as to revert the cancer phenotype to a normal state. Despite promising therapeutic potential in hematopoietic malignancies, it is not clear why epigenetic inhibitors exert less efficacy in solid tumours. Currently, drugs targeting epigenetic regulators directly in CRC are missing. Resistant mechanisms need to be explored to provide a better opportunity for the development of epigenetic drugs in solid cancers.

## 7. Conclusions

Canonical Wnt signalling regulates multiple events involved in embryogenesis and adult homeostasis. In the adult intestine, the Wnt pathway is activated at the bottom of the crypt to maintain intestinal stem cell compartments. However, dysregulation of the Wnt pathway plays a primary role in driving colon cancer cell growth, invasion and survival. While the loss of APC and its consequence in leading to β-catenin accumulation in CRC is well described, mounting data highlight the importance of regulatory networks within the nucleus essential to mediate β-catenin activity. In this review, we have catalogued the main nuclear effectors of β-catenin activity in both tumourigenesis and normal homeostatic control. In addition to their diversity and complexity, as we have detailed, how some nuclear regulators can switch roles from positive to negative effectors based on their conformation (e.g., CtBP), multimolecular assembly (e.g., BRG1), subcellular localisation (e.g., YAP), enzyme activity (e.g., CDK8) or specific tissue distribution (e.g., SIRT1). Furthermore, as we have shown, regulatory event at the level of chromatin configuration and structure expands the ability to control Wnt signalling at a functional and contextual level. Indeed, as illustrated in a number of examples in this review, the ability of regulators to act as activators or repressors may be distinctly contextual on whether the Wnt signal is maintaining gut homeostasis or driving tumourigenic outputs.

Despite intense efforts, clinically approved drugs targeting this pathway are lacking, with a dearth of inhibitors in the clinical trial phases. One explanation centers on the difficulty of untangling Wnt signalling in normal tissue homeostasis from its role in cancer growth. This is underscored by the fact that Wnt pathway inhibitors have generally been found to exhibit toxic effects associated with their on-target activity on normal Wnt pathway signalling. The emerging theme of contextual effect in relation to the ability of nuclear regulators to modulate β-catenin activity offers hope in this regard. As we have illustrated, a number of nuclear β-catenin effectors seem to act in a cancer- or disease-specific manner, raising hope that their targeting may offer a more effective therapeutic window. Conversely, the plasticity of some nuclear β-catenin effectors with respect to their temporal, spatial or compositional features highlights challenges in effectively targeting components which, for example, may actively switch between oncoprotein and tumour suppressor roles. Clearly, additional studies are necessary to understand better the mechanistic underpinnings that may explain this observed plasticity. Indeed, the general concept of proteins changing shape and function to coordinate signalling pathways in response to specific stimuli is well described. As in other systems, a deeper and more comprehensive characterisation of the protein networks which regulate β-catenin transcription will eventually drive the identification of attractive therapeutic targets. In sum, despite the passing of three decades since the initial identification of the Wnt pathway, we have only touched the tip of the iceberg in understanding the intricacies of this signalling pathway. The rewards to be gained from further exploration and characterisation of nuclear β-catenin regulators are immeasurable for the millions of patients affected by Wnt pathway-related diseases every year.

## Figures and Tables

**Table 1:** Summary of regulators influencing nuclear distribution, nuclear abundance and transcriptional activity of β-catenin and TCF/LEF family members.

Regulatory Mechanisms		β-Catenin
Nuclear distribution	pos	KRAS [15]; IQGAP1 [16]; RAPGEF5 [17]; PROX1 [18]; Kinesin-2/IFT-A complex [19]; FOXM1 [20]; CDCP1 [21]; TTPAL/TRIP6 [22] CK2 (Thr393) [23]; RAC1/JNK2 (Ser191, Ser605) [24]; RAC1/PAK1 (Ser675) [25]; BCR-ABL (Tyr86 and Tyr654) [26]; ABL (Tyr 489) [27]
	neg	RHOA [28]; SH3BP4 [29]; CD82 [30]; nuclear APC [31]; nuclear AXIN [32]; RANBP3 [33]; SIRT1 (oncogenic forms of β-catenin) [34]
Nuclear abundance	pos	TCF4 [35]; BCL9 [35]; PIN1 [36]
	neg	NPHP4 [37]; JADE-1 [38]; c-CBL [39,40]
Transcriptional activity	pos	FERMT1 [41]; AKT (Ser552) [42]; RTK (Tyr654) [43]; RTK (Tyr142) [44]
	neg	SIRT1 [34]
Regulatory mechanisms		TCF/LEF family members
Transcriptional activity	pos	TNIK (TCF4) [45]; PIASy (TCF4) [46]
	neg	PIASy (LEF1) [47]; TAK1/NLK (TCF4, LEF1) [48,49]

“Nuclear distribution” lists regulators mediating β-catenin nuclear transport. “Nuclear abundance” involves regulators that promote β-catenin nuclear retention or nuclear degradation. “Transcriptional activity” collects regulators influencing transcriptional activity of β-catenin or TCF/LEF family members. “pos” means positive regulators, and “neg” means negative regulators. Reported modified sites are listed when post-translational modifications occur. We recognise to describe all regulators beyond our scope, and only some well-known and important regulators are summarised here.

**Table 2:** Summary of potential therapeutic strategies and inhibitors targeting dysregulated Wnt pathway.

Therapeutic Strategies	Targets	Inhibitors	Clinical Stage
Targeting the Wnt-receptor complex	Porcupine (PORCN) (important for Wnt secretion)	LGK974 [228]; ETC-159 [229];	LGK974; ETC-159
	WNT-FZD	Vantictumab (OMP-18R5) [230]; Ipafricept (OMP-54F28) [231]; SFRP-1 [232]; Foxy-5 [233]; OTSA-101 [234]	OMP-18R5; OMP-54F28; Foxy-5; OTSA-101
	LRP	Niclosamide [235]; Salinomycin [236]; Monensin [237]	Niclosamide (FDA approved)
	RSPO	OMP-131R10 [238]	OMP-131R10
Targeting the cytoplasmic destruction complex	FZD-DVL	3289-8625 [239]; FJ9 [240]; NSC668036 [241]	None
	Tankyrase (an inhibitor of AXIN)	XAV939 [242]; IWR-1 [243]; JW55/67/64 [244,245]; JW74 [246]; G007-LK [247]; MSC2504877 [248]; RK-287107 [249]; NVP-TNKS656 [250]	None
	CK1	Pyrvinium [251]	Pyrvinium (FDA approved)
	GSK3	LY2090314 [252]	LY2090314
Targeting the nuclear β-catenin-TCF/LEF complex	Β-catenin	MSAB [253]	None
	Β-catenin-TCF4	PKF115-584 and CGP049090; iCRT3, 5, 14; NC043; BC21; LF3; HI-B1 [213,214,215,216,217,218]	None
	Β-catenin-CBP	ICG-001 [219,220,221]; PRI-724 [254]	PRI-724
	Β-catenin-BCL9/9L	SAH-BCL9 [255]; hsBCL9_CT_ [256]	None
	Β-catenin-TRIB3	P2-T3A6 [72]	None
	TNIK	N5355 [257]	None
	CDK8/CDK19	CCT251545 [225]	None

Table 2 summarises the main therapeutic strategies, targets and inhibitors of the Wnt pathway in cancer. The clinical status of each inhibitor is noted. Of note, niclosamide and pyrvinium have been approved by FDA.

## References

[1] Huber A.H., Nelson W.J., Weis W.I. (1997). Three-dimensional structure of the armadillo repeat region of beta-catenin. Cell.

[2] Liu C., Li Y., Semenov M., Han C., Baeg G.H., Tan Y., Zhang Z., Lin X., He X. (2002). Control of beta-catenin phosphorylation/degradation by a dual-kinase mechanism. Cell.

[3] Aberle H., Bauer A., Stappert J., Kispert A., Kemler R. (1997). β-catenin is a target for the ubiquitin–proteasome pathway. EMBO J..

[4] Hart M., Concordet J.P., Lassot I., Albert I., del los Santos R., Durand H., Perret C., Rubinfeld B., Margottin F., Benarous R. (1999). The F-box protein beta-TrCP associates with phosphorylated beta-catenin and regulates its activity in the cell. Curr. Biol..

[5] Wu G., Huang H., Garcia Abreu J., He X. (2009). Inhibition of GSK3 phosphorylation of beta-catenin via phosphorylated PPPSPXS motifs of Wnt coreceptor LRP6. PLoS ONE.

[6] Cselenyi C.S., Jernigan K.K., Tahinci E., Thorne C.A., Lee L.A., Lee E. (2008). LRP6 transduces a canonical Wnt signal independently of Axin degradation by inhibiting GSK3’s phosphorylation of beta-catenin. Proc. Natl. Acad. Sci. USA.

[7] Piao S., Lee S.H., Kim H., Yum S., Stamos J.L., Xu Y., Lee S.J., Lee J., Oh S., Han J.K. (2008). Direct inhibition of GSK3beta by the phosphorylated cytoplasmic domain of LRP6 in Wnt/beta-catenin signaling. PLoS ONE.

[8] Kim S.E., Huang H., Zhao M., Zhang X., Zhang A., Semonov M.V., MacDonald B.T., Zhang X., Garcia Abreu J., Peng L. (2013). Wnt stabilization of beta-catenin reveals principles for morphogen receptor-scaffold assemblies. Science.

[9] Li V.S., Ng S.S., Boersema P.J., Low T.Y., Karthaus W.R., Gerlach J.P., Mohammed S., Heck A.J., Maurice M.M., Mahmoudi T. (2012). Wnt signaling through inhibition of β-catenin degradation in an intact Axin1 complex. Cell.

[10] Schuijers J., Mokry M., Hatzis P., Cuppen E., Clevers H. (2014). Wnt-induced transcriptional activation is exclusively mediated by TCF/LEF. EMBO J..

[11] Novellasdemunt L., Antas P., Li V.S. (2015). Targeting Wnt signaling in colorectal cancer. A Review in the Theme: Cell Signaling: Proteins, Pathways and Mechanisms. Am. J. Physiol. Cell Physiol..

[12] De Lau W., Barker N., Low T.Y., Koo B.-K., Li V.S., Teunissen H., Kujala P., Haegebarth A., Peters P.J., Van De Wetering M. (2011). Lgr5 homologues associate with Wnt receptors and mediate R-spondin signalling. Nature.

[13] Hao H.-X., Xie Y., Zhang Y., Charlat O., Oster E., Avello M., Lei H., Mickanin C., Liu D., Ruffner H. (2012). ZNRF3 promotes Wnt receptor turnover in an R-spondin-sensitive manner. Nature.

[14] Giannakis M., Hodis E., Mu X.J., Yamauchi M., Rosenbluh J., Cibulskis K., Saksena G., Lawrence M.S., Qian Z.R., Nishihara R. (2014). RNF43 is frequently mutated in colorectal and endometrial cancers. Nat. Genet..

[15] Phelps R.A., Chidester S., Dehghanizadeh S., Phelps J., Sandoval I.T., Rai K., Broadbent T., Sarkar S., Burt R.W., Jones D.A. (2009). A two-step model for colon adenoma initiation and progression caused by APC loss. Cell.

[16] Goto T., Sato A., Adachi S., Iemura S., Natsume T., Shibuya H. (2013). IQGAP1 protein regulates nuclear localization of beta-catenin via importin-beta5 protein in Wnt signaling. J. Biol. Chem..

[17] Griffin J.N., Del Viso F., Duncan A.R., Robson A., Hwang W., Kulkarni S., Liu K.J., Khokha M.K. (2018). RAPGEF5 Regulates Nuclear Translocation of beta-Catenin. Dev. Cell..

[18] Liu Y., Ye X., Zhang J.B., Ouyang H., Shen Z., Wu Y., Wang W., Wu J., Tao S., Yang X. (2015). PROX1 promotes hepatocellular carcinoma proliferation and sorafenib resistance by enhancing beta-catenin expression and nuclear translocation. Oncogene.

[19] Vuong L.T., Iomini C., Balmer S., Esposito D., Aaronson S.A., Mlodzik M. (2018). Kinesin-2 and IFT-A act as a complex promoting nuclear localization of beta-catenin during Wnt signalling. Nat. Commun..

[20] Zhang N., Wei P., Gong A., Chiu W.T., Lee H.T., Colman H., Huang H., Xue J., Liu M., Wang Y. (2011). FoxM1 promotes beta-catenin nuclear localization and controls Wnt target-gene expression and glioma tumorigenesis. Cancer Cell.

[21] He Y., Davies C.M., Harrington B.S., Hellmers L., Sheng Y., Broomfield A., McGann T., Bastick K., Zhong L., Wu A. (2020). CDCP1 enhances Wnt signaling in colorectal cancer promoting nuclear localization of β-catenin and E-cadherin. Oncogene.

[22] Gou H., Liang J.Q., Zhang L., Chen H., Zhang Y., Li R., Wang X., Ji J., Tong J.H., To K.F. (2019). TTPAL Promotes Colorectal Tumorigenesis by Stabilizing TRIP6 to Activate Wnt/β-Catenin Signaling. Cancer Res..

[23] Song D.H., Dominguez I., Mizuno J., Kaut M., Mohr S.C., Seldin D.C. (2003). CK2 phosphorylation of the armadillo repeat region of beta-catenin potentiates Wnt signaling. J. Biol. Chem..

[24] Wu X., Tu X., Joeng K.S., Hilton M.J., Williams D.A., Long F. (2008). Rac1 activation controls nuclear localization of beta-catenin during canonical Wnt signaling. Cell.

[25] Zhu G., Wang Y., Huang B., Liang J., Ding Y., Xu A., Wu W. (2012). A Rac1/PAK1 cascade controls beta-catenin activation in colon cancer cells. Oncogene.

[26] Coluccia A.M., Vacca A., Dunach M., Mologni L., Redaelli S., Bustos V.H., Benati D., Pinna L.A., Gambacorti-Passerini C. (2007). Bcr-Abl stabilizes beta-catenin in chronic myeloid leukemia through its tyrosine phosphorylation. EMBO J..

[27] Rhee J., Buchan T., Zukerberg L., Lilien J., Balsamo J. (2007). Cables links Robo-bound Abl kinase to N-cadherin-bound beta-catenin to mediate Slit-induced modulation of adhesion and transcription. Nat. Cell Biol..

[28] Rodrigues P., Macaya I., Bazzocco S., Mazzolini R., Andretta E., Dopeso H., Mateo-Lozano S., Bilic J., Carton-Garcia F., Nieto R. (2014). RHOA inactivation enhances Wnt signalling and promotes colorectal cancer. Nat. Commun..

[29] Antas P., Novellasdemunt L., Kucharska A., Massie I., Carvalho J., Oukrif D., Nye E., Novelli M., Li V.S.W. (2019). SH3BP4 Regulates Intestinal Stem Cells and Tumorigenesis by Modulating beta-Catenin Nuclear Localization. Cell Rep..

[30] Lee M.S., Byun H.J., Lee J., Jeoung D.I., Kim Y.M., Lee H. (2018). Tetraspanin CD82 represses Sp1-mediated Snail expression and the resultant E-cadherin expression interrupts nuclear signaling of beta-catenin by increasing its membrane localization. Cell. Signal..

[31] Neufeld K.L., Zhang F., Cullen B.R., White R.L. (2000). APC-mediated downregulation of beta-catenin activity involves nuclear sequestration and nuclear export. EMBO Rep..

[32] Cong F., Varmus H. (2004). Nuclear-cytoplasmic shuttling of Axin regulates subcellular localization of beta-catenin. Proc. Natl. Acad. Sci. USA.

[33] Hendriksen J., Fagotto F., van der Velde H., van Schie M., Noordermeer J., Fornerod M. (2005). RanBP3 enhances nuclear export of active (beta)-catenin independently of CRM1. J. Cell Biol..

[34] Firestein R., Blander G., Michan S., Oberdoerffer P., Ogino S., Campbell J., Bhimavarapu A., Luikenhuis S., de Cabo R., Fuchs C. (2008). The SIRT1 deacetylase suppresses intestinal tumorigenesis and colon cancer growth. PLoS ONE.

[35] Krieghoff E., Behrens J., Mayr B. (2006). Nucleo-cytoplasmic distribution of beta-catenin is regulated by retention. J. Cell Sci..

[36] Shin H.R., Islam R., Yoon W.J., Lee T., Cho Y.D., Bae H.S., Kim B.S., Woo K.M., Baek J.H., Ryoo H.M. (2016). Pin1-mediated Modification Prolongs the Nuclear Retention of beta-Catenin in Wnt3a-induced Osteoblast Differentiation. J. Biol. Chem..

[37] Borgal L., Habbig S., Hatzold J., Liebau M.C., Dafinger C., Sacarea I., Hammerschmidt M., Benzing T., Schermer B. (2012). The ciliary protein nephrocystin-4 translocates the canonical Wnt regulator Jade-1 to the nucleus to negatively regulate beta-catenin signaling. J. Biol. Chem..

[38] Chitalia V.C., Foy R.L., Bachschmid M.M., Zeng L., Panchenko M.V., Zhou M.I., Bharti A., Seldin D.C., Lecker S.H., Dominguez I. (2008). Jade-1 inhibits Wnt signalling by ubiquitylating beta-catenin and mediates Wnt pathway inhibition by pVHL. Nat. Cell Biol..

[39] Chitalia V., Shivanna S., Martorell J., Meyer R., Edelman E., Rahimi N. (2013). c-Cbl, a ubiquitin E3 ligase that targets active beta-catenin: A novel layer of Wnt signaling regulation. J. Biol. Chem..

[40] Shivanna S., Harrold I., Shashar M., Meyer R., Kiang C., Francis J., Zhao Q., Feng H., Edelman E.R., Rahimi N. (2015). The c-Cbl ubiquitin ligase regulates nuclear beta-catenin and angiogenesis by its tyrosine phosphorylation mediated through the Wnt signaling pathway. J. Biol. Chem..

[41] Liu C.C., Cai D.L., Sun F., Wu Z.H., Yue B., Zhao S.L., Wu X.S., Zhang M., Zhu X.W., Peng Z.H. (2017). FERMT1 mediates epithelial-mesenchymal transition to promote colon cancer metastasis via modulation of beta-catenin transcriptional activity. Oncogene.

[42] Fang D., Hawke D., Zheng Y., Xia Y., Meisenhelder J., Nika H., Mills G.B., Kobayashi R., Hunter T., Lu Z. (2007). Phosphorylation of beta-catenin by AKT promotes beta-catenin transcriptional activity. J. Biol. Chem..

[43] Van Veelen W., Le N.H., Helvensteijn W., Blonden L., Theeuwes M., Bakker E.R., Franken P.F., van Gurp L., Meijlink F., van der Valk M.A. (2011). Beta-catenin tyrosine 654 phosphorylation increases Wnt signalling and intestinal tumorigenesis. Gut.

[44] Brembeck F.H., Schwarz-Romond T., Bakkers J., Wilhelm S., Hammerschmidt M., Birchmeier W. (2004). Essential role of BCL9-2 in the switch between β-catenin’s adhesive and transcriptional functions. Genes Dev..

[45] Mahmoudi T., Li V.S., Ng S.S., Taouatas N., Vries R.G., Mohammed S., Heck A.J., Clevers H. (2009). The kinase TNIK is an essential activator of Wnt target genes. EMBO J..

[46] Yamamoto H., Ihara M., Matsuura Y., Kikuchi A. (2003). Sumoylation is involved in beta-catenin-dependent activation of Tcf-4. EMBO J..

[47] Sachdev S., Bruhn L., Sieber H., Pichler A., Melchior F., Grosschedl R. (2001). PIASy, a nuclear matrix-associated SUMO E3 ligase, represses LEF1 activity by sequestration into nuclear bodies. Genes Dev..

[48] Ishitani T., Ninomiya-Tsuji J., Nagai S., Nishita M., Meneghini M., Barker N., Waterman M., Bowerman B., Clevers H., Shibuya H. (1999). The TAK1-NLK-MAPK-related pathway antagonizes signalling between beta-catenin and transcription factor TCF. Nature.

[49] Ishitani T., Ninomiya-Tsuji J., Matsumoto K. (2003). Regulation of lymphoid enhancer factor 1/T-cell factor by mitogen-activated protein kinase-related Nemo-like kinase-dependent phosphorylation in Wnt/beta-catenin signaling. Mol. Cell Biol..

[50] Nusse R., Clevers H. (2017). Wnt/beta-Catenin Signaling, Disease, and Emerging Therapeutic Modalities. Cell.

[51] Sansom O.J., Meniel V.S., Muncan V., Phesse T.J., Wilkins J.A., Reed K.R., Vass J.K., Athineos D., Clevers H., Clarke A.R. (2007). Myc deletion rescues Apc deficiency in the small intestine. Nature.

[52] Scholz B.A., Sumida N., de Lima C.D.M., Chachoua I., Martino M., Tzelepis I., Nikoshkov A., Zhao H., Mehmood R., Sifakis E.G. (2019). WNT signaling and AHCTF1 promote oncogenic MYC expression through super-enhancer-mediated gene gating. Nat. Genet..

[53] Dow L.E., O’Rourke K.P., Simon J., Tschaharganeh D.F., van Es J.H., Clevers H., Lowe S.W. (2015). Apc Restoration Promotes Cellular Differentiation and Reestablishes Crypt Homeostasis in Colorectal Cancer. Cell.

[54] Torres M.A., Yang-Snyder J.A., Purcell S.M., DeMarais A.A., McGrew L.L., Moon R.T. (1996). Activities of the Wnt-1 class of secreted signaling factors are antagonized by the Wnt-5A class and by a dominant negative cadherin in early Xenopus development. J. Cell Biol..

[55] Dong X., Liao W., Zhang L., Tu X., Hu J., Chen T., Dai X., Xiong Y., Liang W., Ding C. (2017). RSPO2 suppresses colorectal cancer metastasis by counteracting the Wnt5a/Fzd7-driven noncanonical Wnt pathway. Cancer Lett..

[56] Voloshanenko O., Schwartz U., Kranz D., Rauscher B., Linnebacher M., Augustin I., Boutros M. (2018). β-catenin-independent regulation of Wnt target genes by RoR2 and ATF2/ATF4 in colon cancer cells. Sci. Rep..

[57] Lee J.M., Kim I.S., Kim H., Lee J.S., Kim K., Yim H.Y., Jeong J., Kim J.H., Kim J.Y., Lee H. (2010). RORalpha attenuates Wnt/beta-catenin signaling by PKCalpha-dependent phosphorylation in colon cancer. Mol. Cell.

[58] Flores-Hernández E., Velázquez D.M., Castañeda-Patlán M.C., Fuentes-García G., Fonseca-Camarillo G., Yamamoto-Furusho J.K., Romero-Avila M.T., García-Sáinz J.A., Robles-Flores M. (2020). Canonical and non-canonical Wnt signaling are simultaneously activated by Wnts in colon cancer cells. Cell. Signal..

[59] Mosimann C., Hausmann G., Basler K. (2009). Beta-catenin hits chromatin: Regulation of Wnt target gene activation. Nat. Rev. Mol. Cell Biol..

[60] Fiedler M., Sanchez-Barrena M.J., Nekrasov M., Mieszczanek J., Rybin V., Muller J., Evans P., Bienz M. (2008). Decoding of methylated histone H3 tail by the Pygo-BCL9 Wnt signaling complex. Mol. Cell..

[61] Van Tienen L.M., Mieszczanek J., Fiedler M., Rutherford T.J., Bienz M. (2017). Constitutive scaffolding of multiple Wnt enhanceosome components by Legless/BCL9. Elife.

[62] Sustmann C., Flach H., Ebert H., Eastman Q., Grosschedl R. (2008). Cell-type-specific function of BCL9 involves a transcriptional activation domain that synergizes with beta-catenin. Mol. Cell Biol..

[63] Jiang M., Kang Y., Sewastianik T., Wang J., Tanton H., Alder K., Dennis P., Xin Y., Wang Z., Liu R. (2020). BCL9 provides multi-cellular communication properties in colorectal cancer by interacting with paraspeckle proteins. Nat. Commun..

[64] Thompson B., Townsley F., Rosin-Arbesfeld R., Musisi H., Bienz M. (2002). A new nuclear component of the Wnt signalling pathway. Nat. Cell Biol..

[65] Perissi V., Aggarwal A., Glass C.K., Rose D.W., Rosenfeld M.G. (2004). A corepressor/coactivator exchange complex required for transcriptional activation by nuclear receptors and other regulated transcription factors. Cell.

[66] Li J., Wang C.Y. (2008). TBL1-TBLR1 and beta-catenin recruit each other to Wnt target-gene promoter for transcription activation and oncogenesis. Nat. Cell Biol..

[67] Jung H.Y., Jun S., Lee M., Kim H.C., Wang X., Ji H., McCrea P.D., Park J.I. (2013). PAF and EZH2 induce Wnt/beta-catenin signaling hyperactivation. Mol. Cell..

[68] Chen Y., Li Y., Xue J., Gong A., Yu G., Zhou A., Lin K., Zhang S., Zhang N., Gottardi C.J. (2016). Wnt-induced deubiquitination FoxM1 ensures nucleus beta-catenin transactivation. EMBO J..

[69] Kang D.W., Lee S.H., Yoon J.W., Park W.S., Choi K.Y., Min do S. (2010). Phospholipase D1 drives a positive feedback loop to reinforce the Wnt/beta-catenin/TCF signaling axis. Cancer Res..

[70] Kang D.W., Lee B.H., Suh Y.A., Choi Y.S., Jang S.J., Kim Y.M., Choi K.Y., Min D.S. (2017). Phospholipase D1 Inhibition Linked to Upregulation of ICAT Blocks Colorectal Cancer Growth Hyperactivated by Wnt/beta-Catenin and PI3K/Akt Signaling. Clin. Cancer Res. Off. J. Am. Assoc. Cancer Res..

[71] Miyoshi N., Ishii H., Mimori K., Takatsuno Y., Kim H., Hirose H., Sekimoto M., Doki Y., Mori M. (2009). Abnormal expression of TRIB3 in colorectal cancer: A novel marker for prognosis. Br. J. Cancer..

[72] Hua F., Shang S., Yang Y.W., Zhang H.Z., Xu T.L., Yu J.J., Zhou D.D., Cui B., Li K., Lv X.X. (2019). TRIB3 Interacts With beta-Catenin and TCF4 to Increase Stem Cell Features of Colorectal Cancer Stem Cells and Tumorigenesis. Gastroenterology.

[73] Mir R., Pradhan S.J., Patil P., Mulherkar R., Galande S. (2016). Wnt/beta-catenin signaling regulated SATB1 promotes colorectal cancer tumorigenesis and progression. Oncogene.

[74] Jamora C., DasGupta R., Kocieniewski P., Fuchs E. (2003). Links between signal transduction, transcription and adhesion in epithelial bud development. Nature.

[75] Piepenburg O., Vorbruggen G., Jackle H. (2000). Drosophila segment borders result from unilateral repression of hedgehog activity by wingless signaling. Mol. Cell..

[76] Blauwkamp T.A., Chang M.V., Cadigan K.M. (2008). Novel TCF-binding sites specify transcriptional repression by Wnt signalling. EMBO J..

[77] Tago K., Nakamura T., Nishita M., Hyodo J., Nagai S., Murata Y., Adachi S., Ohwada S., Morishita Y., Shibuya H. (2000). Inhibition of Wnt signaling by ICAT, a novel beta-catenin-interacting protein. Genes Dev..

[78] Daniels D.L., Weis W.I. (2002). ICAT inhibits beta-catenin binding to Tcf/Lef-family transcription factors and the general coactivator p300 using independent structural modules. Mol. Cell.

[79] Ji L., Lu B., Wang Z., Yang Z., Reece-Hoyes J., Russ C., Xu W., Cong F. (2018). Identification of ICAT as an APC Inhibitor, Revealing Wnt-Dependent Inhibition of APC-Axin Interaction. Mol. Cell.

[80] Wang S., Dong Y., Zhang Y., Wang X., Xu L., Yang S., Li X., Dong H., Xu L., Su L. (2015). DACT2 is a functional tumor suppressor through inhibiting Wnt/beta-catenin pathway and associated with poor survival in colon cancer. Oncogene.

[81] Takemaru K., Yamaguchi S., Lee Y.S., Zhang Y., Carthew R.W., Moon R.T. (2003). Chibby, a nuclear beta-catenin-associated antagonist of the Wnt/Wingless pathway. Nature.

[82] Li F.Q., Mofunanya A., Harris K., Takemaru K. (2008). Chibby cooperates with 14-3-3 to regulate beta-catenin subcellular distribution and signaling activity. J. Cell Biol..

[83] Brannon M., Brown J.D., Bates R., Kimelman D., Moon R.T. (1999). XCtBP is a XTcf-3 co-repressor with roles throughout Xenopus development. Development.

[84] Valenta T., Lukas J., Korinek V. (2003). HMG box transcription factor TCF-4’s interaction with CtBP1 controls the expression of the Wnt target Axin2/Conductin in human embryonic kidney cells. Nucleic Acids Res..

[85] Hamada F., Bienz M. (2004). The APC tumor suppressor binds to C-terminal binding protein to divert nuclear beta-catenin from TCF. Dev. Cell..

[86] Sierra J., Yoshida T., Joazeiro C.A., Jones K.A. (2006). The APC tumor suppressor counteracts β-catenin activation and H3K4 methylation at Wnt target genes. Genes Dev..

[87] Fang M., Li J., Blauwkamp T., Bhambhani C., Campbell N., Cadigan K.M. (2006). C-terminal-binding protein directly activates and represses Wnt transcriptional targets in Drosophila. EMBO J..

[88] Bhambhani C., Chang J.L., Akey D.L., Cadigan K.M. (2011). The oligomeric state of CtBP determines its role as a transcriptional co-activator and co-repressor of Wingless targets. EMBO J..

[89] Huang J., Wu S., Barrera J., Matthews K., Pan D. (2005). The Hippo signaling pathway coordinately regulates cell proliferation and apoptosis by inactivating Yorkie, the Drosophila Homolog of YAP. Cell.

[90] Li J., Chen X., Ding X., Cheng Y., Zhao B., Lai Z.C., Al Hezaimi K., Hakem R., Guan K.L., Wang C.-Y. (2013). LATS2 suppresses oncogenic Wnt signaling by disrupting beta-catenin/BCL9 interaction. Cell Rep..

[91] Zhang C., Cho K., Huang Y., Lyons J.P., Zhou X., Sinha K., McCrea P.D., de Crombrugghe B. (2008). Inhibition of Wnt signaling by the osteoblast-specific transcription factor Osterix. Proc. Natl. Acad. Sci. USA.

[92] Tang W., Dodge M., Gundapaneni D., Michnoff C., Roth M., Lum L. (2008). A genome-wide RNAi screen for Wnt/beta-catenin pathway components identifies unexpected roles for TCF transcription factors in cancer. Proc. Natl. Acad. Sci. USA.

[93] Angus-Hill M.L., Elbert K.M., Hidalgo J., Capecchi M.R. (2011). T-cell factor 4 functions as a tumor suppressor whose disruption modulates colon cell proliferation and tumorigenesis. Proc. Natl. Acad. Sci. USA.

[94] Rosenbluh J., Nijhawan D., Cox A.G., Li X., Neal J.T., Schafer E.J., Zack T.I., Wang X., Tsherniak A., Schinzel A.C. (2012). β-Catenin-driven cancers require a YAP1 transcriptional complex for survival and tumorigenesis. Cell.

[95] Rosenbluh J., Mercer J., Shrestha Y., Oliver R., Tamayo P., Doench J.G., Tirosh I., Piccioni F., Hartenian E., Horn H. (2016). Genetic and Proteomic Interrogation of Lower Confidence Candidate Genes Reveals Signaling Networks in beta-Catenin-Active Cancers. Cell Syst..

[96] Li Q., Sun Y., Jarugumilli G.K., Liu S., Dang K., Cotton J.L., Xiol J., Chan P.Y., DeRan M., Ma L. (2020). Lats1/2 Sustain Intestinal Stem Cells and Wnt Activation through TEAD-Dependent and Independent Transcription. Cell Stem Cell.

[97] Barry E.R., Morikawa T., Butler B.L., Shrestha K., de la Rosa R., Yan K.S., Fuchs C.S., Magness S.T., Smits R., Ogino S. (2013). Restriction of intestinal stem cell expansion and the regenerative response by YAP. Nature.

[98] Imajo M., Miyatake K., Iimura A., Miyamoto A., Nishida E. (2012). A molecular mechanism that links Hippo signalling to the inhibition of Wnt/beta-catenin signalling. EMBO J..

[99] Azzolin L., Panciera T., Soligo S., Enzo E., Bicciato S., Dupont S., Bresolin S., Frasson C., Basso G., Guzzardo V. (2014). YAP/TAZ incorporation in the beta-catenin destruction complex orchestrates the Wnt response. Cell.

[100] Jiao S., Li C., Hao Q., Miao H., Zhang L., Li L., Zhou Z. (2017). VGLL4 targets a TCF4-TEAD4 complex to coregulate Wnt and Hippo signalling in colorectal cancer. Nat. Commun..

[101] Kelly K.F., Ng D.Y., Jayakumaran G., Wood G.A., Koide H., Doble B.W. (2011). Beta-catenin enhances Oct-4 activity and reinforces pluripotency through a TCF-independent mechanism. Cell Stem Cell.

[102] Abu-Remaileh M., Gerson A., Farago M., Nathan G., Alkalay I., Zins Rousso S., Gur M., Fainsod A., Bergman Y. (2010). Oct-3/4 regulates stem cell identity and cell fate decisions by modulating Wnt/beta-catenin signalling. EMBO J..

[103] Harris A.L. (2002). Hypoxia—A key regulatory factor in tumour growth. Nat. Rev. Cancer.

[104] Hoogeboom D., Essers M.A., Polderman P.E., Voets E., Smits L.M., Burgering B.M. (2008). Interaction of FOXO with beta-catenin inhibits beta-catenin/T cell factor activity. J. Biol. Chem..

[105] Essers M.A., de Vries-Smits L.M., Barker N., Polderman P.E., Burgering B.M., Korswagen H.C. (2005). Functional interaction between beta-catenin and FOXO in oxidative stress signaling. Science.

[106] Kaidi A., Williams A.C., Paraskeva C. (2007). Interaction between beta-catenin and HIF-1 promotes cellular adaptation to hypoxia. Nat. Cell Biol..

[107] Doumpas N., Lampart F., Robinson M.D., Lentini A., Nestor C.E., Cantu C., Basler K. (2019). TCF/LEF dependent and independent transcriptional regulation of Wnt/beta-catenin target genes. EMBO J..

[108] Bastide P., Darido C., Pannequin J., Kist R., Robine S., Marty-Double C., Bibeau F., Scherer G., Joubert D., Hollande F. (2007). Sox9 regulates cell proliferation and is required for Paneth cell differentiation in the intestinal epithelium. J. Cell Biol..

[109] Mori-Akiyama Y., van den Born M., van Es J.H., Hamilton S.R., Adams H.P., Zhang J., Clevers H., de Crombrugghe B. (2007). SOX9 is required for the differentiation of paneth cells in the intestinal epithelium. Gastroenterology.

[110] Blache P., van de Wetering M., Duluc I., Domon C., Berta P., Freund J.N., Clevers H., Jay P. (2004). SOX9 is an intestine crypt transcription factor, is regulated by the Wnt pathway, and represses the CDX2 and MUC2 genes. J. Cell Biol..

[111] Darido C., Buchert M., Pannequin J., Bastide P., Zalzali H., Mantamadiotis T., Bourgaux J.F., Garambois V., Jay P., Blache P. (2008). Defective claudin-7 regulation by Tcf-4 and Sox-9 disrupts the polarity and increases the tumorigenicity of colorectal cancer cells. Cancer Res..

[112] Lü B., Fang Y., Xu J., Wang L., Xu F., Xu E., Huang Q., Lai M. (2008). Analysis of SOX9 expression in colorectal cancer. Am. J. Clin. Pathol..

[113] Matheu A., Collado M., Wise C., Manterola L., Cekaite L., Tye A.J., Canamero M., Bujanda L., Schedl A., Cheah K.S. (2012). Oncogenicity of the developmental transcription factor Sox9. Cancer Res..

[114] Shi Z., Chiang C.I., Labhart P., Zhao Y., Yang J., Mistretta T.A., Henning S.J., Maity S.N., Mori-Akiyama Y. (2015). Context-specific role of SOX9 in NF-Y mediated gene regulation in colorectal cancer cells. Nucleic Acids Res..

[115] Whissell G., Montagni E., Martinelli P., Hernando-Momblona X., Sevillano M., Jung P., Cortina C., Calon A., Abuli A., Castells A. (2014). The transcription factor GATA6 enables self-renewal of colon adenoma stem cells by repressing BMP gene expression. Nat. Cell Biol..

[116] Tsuji S., Kawasaki Y., Furukawa S., Taniue K., Hayashi T., Okuno M., Hiyoshi M., Kitayama J., Akiyama T. (2014). The miR-363-GATA6-Lgr5 pathway is critical for colorectal tumourigenesis. Nat. Commun..

[117] Poss Z.C., Ebmeier C.C., Taatjes D.J. (2013). The Mediator complex and transcription regulation. Crit. Rev. Biochem. Mol. Biol..

[118] Jeronimo C., Robert F. (2017). The Mediator Complex: At the Nexus of RNA Polymerase II Transcription. Trends Cell Biol..

[119] Manning G., Whyte D.B., Martinez R., Hunter T., Sudarsanam S. (2002). The protein kinase complement of the human genome. Science.

[120] Malumbres M. (2014). Cyclin-dependent kinases. Genome Biol..

[121] Allen B.L., Taatjes D.J. (2015). The Mediator complex: A central integrator of transcription. Nat. Rev. Mol. Cell Biol..

[122] Klatt F., Leitner A., Kim I.V., Ho-Xuan H., Schneider E.V., Langhammer F., Weinmann R., Muller M.R., Huber R., Meister G. (2020). A precisely positioned MED12 activation helix stimulates CDK8 kinase activity. Proc. Natl. Acad. Sci. USA.

[123] Galbraith M.D., Allen M.A., Bensard C.L., Wang X., Schwinn M.K., Qin B., Long H.W., Daniels D.L., Hahn W.C., Dowell R.D. (2013). HIF1A employs CDK8-mediator to stimulate RNAPII elongation in response to hypoxia. Cell.

[124] Chen M., Liang J., Ji H., Yang Z., Altilia S., Hu B., Schronce A., McDermott M.S., Schools G.P., Lim C.-u. (2017). CDK8/19 Mediator kinases potentiate induction of transcription by NFκB. Proc. Natl. Acad. Sci. USA.

[125] Alarcon C., Zaromytidou A.I., Xi Q., Gao S., Yu J., Fujisawa S., Barlas A., Miller A.N., Manova-Todorova K., Macias M.J. (2009). Nuclear CDKs drive Smad transcriptional activation and turnover in BMP and TGF-beta pathways. Cell.

[126] Roninson I.B., Gyorffy B., Mack Z.T., Shtil A.A., Shtutman M.S., Chen M., Broude E.V. (2019). Identifying Cancers Impacted by CDK8/19. Cells.

[127] Firestein R., Bass A.J., Kim S.Y., Dunn I.F., Silver S.J., Guney I., Freed E., Ligon A.H., Vena N., Ogino S. (2008). CDK8 is a colorectal cancer oncogene that regulates beta-catenin activity. Nature.

[128] Carrera I., Janody F., Leeds N., Duveau F., Treisman J.E. (2008). Pygopus activates Wingless target gene transcription through the mediator complex subunits Med12 and Med13. Proc. Natl. Acad. Sci. USA.

[129] Kim S., Xu X., Hecht A., Boyer T.G. (2006). Mediator is a transducer of Wnt/beta-catenin signaling. J. Biol. Chem..

[130] Morris E.J., Ji J.Y., Yang F., Di Stefano L., Herr A., Moon N.S., Kwon E.J., Haigis K.M., Naar A.M., Dyson N.J. (2008). E2F1 represses beta-catenin transcription and is antagonized by both pRB and CDK8. Nature.

[131] Wu Z., Zheng S., Li Z., Tan J., Yu Q. (2011). E2F1 suppresses Wnt/beta-catenin activity through transactivation of beta-catenin interacting protein ICAT. Oncogene.

[132] Zhao J., Ramos R., Demma M. (2013). CDK8 regulates E2F1 transcriptional activity through S375 phosphorylation. Oncogene.

[133] Zhou J., Zeng Y., Cui L., Chen X., Stauffer S., Wang Z., Yu F., Lele S.M., Talmon G.A., Black A.R. (2018). Zyxin promotes colon cancer tumorigenesis in a mitotic phosphorylation-dependent manner and through CDK8-mediated YAP activation. Proc. Natl. Acad. Sci. USA.

[134] Kornberg R.D. (1974). Chromatin structure: A repeating unit of histones and DNA. Science.

[135] Luger K., Mader A.W., Richmond R.K., Sargent D.F., Richmond T.J. (1997). Crystal structure of the nucleosome core particle at 2.8 A resolution. Nature.

[136] Kornberg R.D., Lorch Y. (1999). Twenty-five years of the nucleosome, fundamental particle of the eukaryote chromosome. Cell.

[137] Kingston R.E., Narlikar G.J. (1999). ATP-dependent remodeling and acetylation as regulators of chromatin fluidity. Genes Dev..

[138] Dimitrova E., Turberfield A.H., Klose R.J. (2015). Histone demethylases in chromatin biology and beyond. EMBO Rep..

[139] Santos-Rosa H., Caldas C. (2005). Chromatin modifier enzymes, the histone code and cancer. Eur. J. Cancer.

[140] Esteller M. (2006). Epigenetics provides a new generation of oncogenes and tumour-suppressor genes. Br. J. Cancer.

[141] Baylin S.B., Jones P.A. (2011). A decade of exploring the cancer epigenome—biological and translational implications. Nat. Rev. Cancer.

[142] Cheung W.L., Briggs S.D., Allis C.D. (2000). Acetylation and chromosomal functions. Curr. Opin. Cell Biol..

[143] Kalkhoven E. (2004). CBP and p300: HATs for different occasions. Biochem. Pharmacol..

[144] Sterner D.E., Berger S.L. (2000). Acetylation of histones and transcription-related factors. Microbiol. Mol. Biol. Rev..

[145] Waltzer L., Bienz M. (1998). Drosophila CBP represses the transcription factor TCF to antagonize Wingless signalling. Nature.

[146] Li J., Sutter C., Parker D.S., Blauwkamp T., Fang M., Cadigan K.M. (2007). CBP/p300 are bimodal regulators of Wnt signaling. EMBO J..

[147] Hecht A., Vleminckx K., Stemmler M.P., van Roy F., Kemler R. (2000). The p300/CBP acetyltransferases function as transcriptional coactivators of beta-catenin in vertebrates. EMBO J..

[148] Miyagishi M., Fujii R., Hatta M., Yoshida E., Araya N., Nagafuchi A., Ishihara S., Nakajima T., Fukamizu A. (2000). Regulation of Lef-mediated transcription and p53-dependent pathway by associating beta-catenin with CBP/p300. J. Biol Chem..

[149] Takemaru K.I., Moon R.T. (2000). The transcriptional coactivator CBP interacts with beta-catenin to activate gene expression. J. Cell Biol..

[150] Sun Y., Kolligs F.T., Hottiger M.O., Mosavin R., Fearon E.R., Nabel G.J. (2000). Regulation of beta-catenin transformation by the p300 transcriptional coactivator. Proc. Natl. Acad. Sci. USA.

[151] Ma H., Nguyen C., Lee K.S., Kahn M. (2005). Differential roles for the coactivators CBP and p300 on TCF/beta-catenin-mediated survivin gene expression. Oncogene.

[152] Miyabayashi T., Teo J.L., Yamamoto M., McMillan M., Nguyen C., Kahn M. (2007). Wnt/beta-catenin/CBP signaling maintains long-term murine embryonic stem cell pluripotency. Proc. Natl. Acad. Sci. USA.

[153] Teo J.L., Kahn M. (2010). The Wnt signaling pathway in cellular proliferation and differentiation: A tale of two coactivators. Adv. Drug Deliv. Rev..

[154] Wolf D., Rodova M., Miska E.A., Calvet J.P., Kouzarides T. (2002). Acetylation of beta-catenin by CREB-binding protein (CBP). J. Biol. Chem..

[155] Levy L., Wei Y., Labalette C., Wu Y., Renard C.A., Buendia M.A., Neuveut C. (2004). Acetylation of beta-catenin by p300 regulates beta-catenin-Tcf4 interaction. Mol. Cell Biol..

[156] Rieger M.E., Zhou B., Solomon N., Sunohara M., Li C., Nguyen C., Liu Y., Pan J.H., Minoo P., Crandall E.D. (2016). p300/beta-Catenin Interactions Regulate Adult Progenitor Cell Differentiation Downstream of WNT5a/Protein Kinase C (PKC). J. Biol. Chem..

[157] Ge X., Jin Q., Zhang F., Yan T., Zhai Q. (2009). PCAF acetylates β-catenin and improves its stability. Mol. Biol. Cell.

[158] Cai Y., Jin J., Tomomori-Sato C., Sato S., Sorokina I., Parmely T.J., Conaway R.C., Conaway J.W. (2003). Identification of new subunits of the multiprotein mammalian TRRAP/TIP60-containing histone acetyltransferase complex. J. Biol. Chem..

[159] Feng Y., Lee N., Fearon E.R. (2003). TIP49 regulates beta-catenin-mediated neoplastic transformation and T-cell factor target gene induction via effects on chromatin remodeling. Cancer Res..

[160] Bauer A., Chauvet S., Huber O., Usseglio F., Rothbächer U., Aragnol D., Kemler R., Pradel J. (2000). Pontin52 and Reptin52 function as antagonistic regulators of β-catenin signalling activity. EMBO J..

[161] Kim J.H., Kim B., Cai L., Choi H.J., Ohgi K.A., Tran C., Chen C., Chung C.H., Huber O., Rose D.W. (2005). Transcriptional regulation of a metastasis suppressor gene by Tip60 and beta-catenin complexes. Nature.

[162] Billin A.N., Thirlwell H., Ayer D.E. (2000). Beta-catenin-histone deacetylase interactions regulate the transition of LEF1 from a transcriptional repressor to an activator. Mol. Cell Biol..

[163] Ye F., Chen Y., Hoang T., Montgomery R.L., Zhao X.H., Bu H., Hu T., Taketo M.M., van Es J.H., Clevers H. (2009). HDAC1 and HDAC2 regulate oligodendrocyte differentiation by disrupting the beta-catenin-TCF interaction. Nat. Neurosci..

[164] Bhaumik S.R., Smith E., Shilatifard A. (2007). Covalent modifications of histones during development and disease pathogenesis. Nat. Struct. Mol. Biol..

[165] Shilatifard A. (2008). Molecular implementation and physiological roles for histone H3 lysine 4 (H3K4) methylation. Curr. Opin. Cell Biol..

[166] Santos-Rosa H., Schneider R., Bannister A.J., Sherriff J., Bernstein B.E., Emre N.C., Schreiber S.L., Mellor J., Kouzarides T. (2002). Active genes are tri-methylated at K4 of histone H3. Nature.

[167] Mohan M., Lin C., Guest E., Shilatifard A. (2010). Licensed to elongate: A molecular mechanism for MLL-based leukaemogenesis. Nat. Rev. Cancer.

[168] Yu B.D., Hanson R.D., Hess J.L., Horning S.E., Korsmeyer S.J. (1998). MLL a mammalian trithorax-group gene, functions as a transcriptional maintenance factor in morphogenesis. Proc. Natl. Acad. Sci. USA.

[169] Hsieh J.J., Cheng E.H., Korsmeyer S.J. (2003). Taspase1: A threonine aspartase required for cleavage of MLL and proper HOX gene expression. Cell.

[170] Hsieh J.J., Ernst P., Erdjument-Bromage H., Tempst P., Korsmeyer S.J. (2003). Proteolytic cleavage of MLL generates a complex of N- and C-terminal fragments that confers protein stability and subnuclear localization. Mol. Cell Biol..

[171] Ayton P.M., Cleary M.L. (2001). Molecular mechanisms of leukemogenesis mediated by MLL fusion proteins. Oncogene.

[172] Krivtsov A.V., Armstrong S.A. (2007). MLL translocations, histone modifications and leukaemia stem-cell development. Nat. Rev. Cancer.

[173] Fang Y., Zhang D., Hu T., Zhao H., Zhao X., Lou Z., He Y., Qin W., Xia J., Zhang X. (2019). KMT2A histone methyltransferase contributes to colorectal cancer development by promoting cathepsin Z transcriptional activation. Cancer Med..

[174] Wend P., Fang L., Zhu Q., Schipper J.H., Loddenkemper C., Kosel F., Brinkmann V., Eckert K., Hindersin S., Holland J.D. (2013). Wnt/beta-catenin signalling induces MLL to create epigenetic changes in salivary gland tumours. EMBO J..

[175] Zhu Q., Fang L., Heuberger J., Kranz A., Schipper J., Scheckenbach K., Vidal R.O., Sunaga-Franze D.Y., Muller M., Wulf-Goldenberg A. (2019). The Wnt-Driven Mll1 Epigenome Regulates Salivary Gland and Head and Neck Cancer. Cell Rep..

[176] Zhou C., Zhang Y., Dai J., Zhou M., Liu M., Wang Y., Chen X.Z., Tang J. (2016). Pygo2 functions as a prognostic factor for glioma due to its up-regulation of H3K4me3 and promotion of MLL1/MLL2 complex recruitment. Sci. Rep..

[177] Salz T., Li G., Kaye F., Zhou L., Qiu Y., Huang S. (2014). hSETD1A regulates Wnt target genes and controls tumor growth of colorectal cancer cells. Cancer Res..

[178] Tan J.Z., Yan Y., Wang X.X., Jiang Y., Xu H.E. (2014). EZH2: Biology, disease, and structure-based drug discovery. Acta Pharmacol. Sin..

[179] Cheng A.S., Lau S.S., Chen Y., Kondo Y., Li M.S., Feng H., Ching A.K., Cheung K.F., Wong H.K., Tong J.H. (2011). EZH2-mediated concordant repression of Wnt antagonists promotes beta-catenin-dependent hepatocarcinogenesis. Cancer Res..

[180] Chen L., Wu Y., Wu Y., Wang Y., Sun L., Li F. (2016). The inhibition of EZH2 ameliorates osteoarthritis development through the Wnt/beta-catenin pathway. Sci. Rep..

[181] Fang J., Feng Q., Ketel C.S., Wang H., Cao R., Xia L., Erdjument-Bromage H., Tempst P., Simon J.A., Zhang Y. (2002). Purification and functional characterization of SET8, a nucleosomal histone H4-lysine 20-specific methyltransferase. Curr. Biol..

[182] Li Z., Nie F., Wang S., Li L. (2011). Histone H4 Lys 20 monomethylation by histone methylase SET8 mediates Wnt target gene activation. Proc. Natl. Acad. Sci. USA.

[183] Yang F., Sun L., Li Q., Han X., Lei L., Zhang H., Shang Y. (2012). SET8 promotes epithelial-mesenchymal transition and confers TWIST dual transcriptional activities. EMBO J..

[184] Kim H.A., Koo B.K., Cho J.H., Kim Y.Y., Seong J., Chang H.J., Oh Y.M., Stange D.E., Park J.G., Hwang D. (2012). Notch1 counteracts WNT/β-catenin signaling through chromatin modification in colorectal cancer. J. Clin. Investig..

[185] Feng Q., Wang H., Ng H.H., Erdjument-Bromage H., Tempst P., Struhl K., Zhang Y. (2002). Methylation of H3-lysine 79 is mediated by a new family of HMTases without a SET domain. Curr. Biol..

[186] Mahmoudi T., Boj S.F., Hatzis P., Li V.S., Taouatas N., Vries R.G., Teunissen H., Begthel H., Korving J., Mohammed S. (2010). The leukemia-associated Mllt10/Af10-Dot1l are Tcf4/beta-catenin coactivators essential for intestinal homeostasis. PLoS Biol..

[187] Mohan M., Herz H.M., Takahashi Y.H., Lin C., Lai K.C., Zhang Y., Washburn M.P., Florens L., Shilatifard A. (2010). Linking H3K79 trimethylation to Wnt signaling through a novel Dot1-containing complex (DotCom). Genes Dev..

[188] Monteagudo S., Cornelis F.M.F., Aznar-Lopez C., Yibmantasiri P., Guns L.A., Carmeliet P., Cailotto F., Lories R.J. (2017). DOT1L safeguards cartilage homeostasis and protects against osteoarthritis. Nat. Commun..

[189] Yang Y., Bedford M.T. (2013). Protein arginine methyltransferases and cancer. Nat. Rev. Cancer.

[190] Karkhanis V., Hu Y.J., Baiocchi R.A., Imbalzano A.N., Sif S. (2011). Versatility of PRMT5-induced methylation in growth control and development. Trends Biochem. Sci..

[191] Chung J., Karkhanis V., Baiocchi R.A., Sif S. (2019). Protein arginine methyltransferase 5 (PRMT5) promotes survival of lymphoma cells via activation of WNT/beta-catenin and AKT/GSK3beta proliferative signaling. J. Biol. Chem..

[192] Jin Y., Zhou J., Xu F., Jin B., Cui L., Wang Y., Du X., Li J., Li P., Ren R. (2016). Targeting methyltransferase PRMT5 eliminates leukemia stem cells in chronic myelogenous leukemia. J. Clin. Investig..

[193] Zhu K., Peng Y., Hu J., Zhan H., Yang L., Gao Q., Jia H., Luo R., Dai Z., Tang Z. (2020). Metadherin-PRMT5 complex enhances the metastasis of hepatocellular carcinoma through the WNT-beta-catenin signaling pathway. Carcinogenesis.

[194] Li J., Yu B., Deng P., Cheng Y., Yu Y., Kevork K., Ramadoss S., Ding X., Li X., Wang C.Y. (2017). KDM3 epigenetically controls tumorigenic potentials of human colorectal cancer stem cells through Wnt/beta-catenin signalling. Nat. Commun..

[195] Peng K., Su G., Ji J., Yang X., Miao M., Mo P., Li M., Xu J., Li W., Yu C. (2018). Histone demethylase JMJD1A promotes colorectal cancer growth and metastasis by enhancing Wnt/beta-catenin signaling. J. Biol. Chem..

[196] Peng K., Kou L., Yu L., Bai C., Li M., Mo P., Li W., Yu C. (2019). Histone Demethylase JMJD2D Interacts With beta-Catenin to Induce Transcription and Activate Colorectal Cancer Cell Proliferation and Tumor Growth in Mice. Gastroenterology.

[197] Huang Z., Li S., Song W., Li X., Li Q., Zhang Z., Han Y., Zhang X., Miao S., Du R. (2013). Lysine-specific demethylase 1 (LSD1/KDM1A) contributes to colorectal tumorigenesis via activation of the Wnt/β-catenin pathway by down-regulating Dickkopf-1 (DKK1) [corrected]. PLoS ONE.

[198] Xue Y., Canman J.C., Lee C.S., Nie Z., Yang D., Moreno G.T., Young M.K., Salmon E.D., Wang W. (2000). The human SWI/SNF-B chromatin-remodeling complex is related to yeast rsc and localizes at kinetochores of mitotic chromosomes. Proc. Natl. Acad. Sci. USA.

[199] Wang W., Xue Y., Zhou S., Kuo A., Cairns B.R., Crabtree G.R. (1996). Diversity and specialization of mammalian SWI/SNF complexes. Genes Dev..

[200] Holstege F.C., Jennings E.G., Wyrick J.J., Lee T.I., Hengartner C.J., Green M.R., Golub T.R., Lander E.S., Young R.A. (1998). Dissecting the regulatory circuitry of a eukaryotic genome. Cell.

[201] Barker N., Hurlstone A., Musisi H., Miles A., Bienz M., Clevers H. (2001). The chromatin remodelling factor Brg-1 interacts with beta-catenin to promote target gene activation. EMBO J..

[202] Wang G., Fu Y., Yang X., Luo X., Wang J., Gong J., Hu J. (2016). Brg-1 targeting of novel miR550a-5p/RNF43/Wnt signaling axis regulates colorectal cancer metastasis. Oncogene.

[203] Park J.I., Venteicher A.S., Hong J.Y., Choi J., Jun S., Shkreli M., Chang W., Meng Z., Cheung P., Ji H. (2009). Telomerase modulates Wnt signalling by association with target gene chromatin. Nature.

[204] Mora-Blanco E.L., Mishina Y., Tillman E.J., Cho Y.J., Thom C.S., Pomeroy S.L., Shao W., Roberts C.W. (2014). Activation of beta-catenin/TCF targets following loss of the tumor suppressor SNF5. Oncogene.

[205] Major M.B., Roberts B.S., Berndt J.D., Marine S., Anastas J., Chung N., Ferrer M., Yi X., Stoick-Cooper C.L., von Haller P.D. (2008). New regulators of Wnt/beta-catenin signaling revealed by integrative molecular screening. Sci. Signal..

[206] Mathur R., Alver B.H., San Roman A.K., Wilson B.G., Wang X., Agoston A.T., Park P.J., Shivdasani R.A., Roberts C.W. (2017). ARID1A loss impairs enhancer-mediated gene regulation and drives colon cancer in mice. Nat. Genet..

[207] Hiramatsu Y., Fukuda A., Ogawa S., Goto N., Ikuta K., Tsuda M., Matsumoto Y., Kimura Y., Yoshioka T., Takada Y. (2019). Arid1a is essential for intestinal stem cells through Sox9 regulation. Proc. Natl. Acad. Sci. USA.

[208] Vasileiou G., Ekici A.B., Uebe S., Zweier C., Hoyer J., Engels H., Behrens J., Reis A., Hadjihannas M.V. (2015). Chromatin-Remodeling-Factor ARID1B Represses Wnt/beta-Catenin Signaling. Am. J. Hum. Genet..

[209] Seshagiri S., Stawiski E.W., Durinck S., Modrusan Z., Storm E.E., Conboy C.B., Chaudhuri S., Guan Y., Janakiraman V., Jaiswal B.S. (2012). Recurrent R-spondin fusions in colon cancer. Nature.

[210] Storm E.E., Durinck S., de Sousa e Melo F., Tremayne J., Kljavin N., Tan C., Ye X., Chiu C., Pham T., Hongo J.A. (2016). Targeting PTPRK-RSPO3 colon tumours promotes differentiation and loss of stem-cell function. Nature.

[211] Michels B.E., Mosa M.H., Grebbin B.M., Yepes D., Darvishi T., Hausmann J., Urlaub H., Zeuzem S., Kvasnicka H.M., Oellerich T. (2019). Human colon organoids reveal distinct physiologic and oncogenic Wnt responses. J. Exp. Med..

[212] Billmann M., Chaudhary V., ElMaghraby M.F., Fischer B., Boutros M. (2018). Widespread Rewiring of Genetic Networks upon Cancer Signaling Pathway Activation. Cell Syst..

[213] Lepourcelet M., Chen Y.-N.P., France D.S., Wang H., Crews P., Petersen F., Bruseo C., Wood A.W., Shivdasani R.A. (2004). Small-molecule antagonists of the oncogenic Tcf/β-catenin protein complex. Cancer Cell.

[214] Gonsalves F.C., Klein K., Carson B.B., Katz S., Ekas L.A., Evans S., Nagourney R., Cardozo T., Brown A.M., DasGupta R. (2011). An RNAi-based chemical genetic screen identifies three small-molecule inhibitors of the Wnt/wingless signaling pathway. Proc. Natl. Acad. Sci. USA.

[215] Wang W., Liu H., Wang S., Hao X., Li L. (2011). A diterpenoid derivative 15-oxospiramilactone inhibits Wnt/beta-catenin signaling and colon cancer cell tumorigenesis. Cell Res..

[216] Tian W., Han X., Yan M., Xu Y., Duggineni S., Lin N., Luo G., Li Y.M., Han X., Huang Z. (2012). Structure-based discovery of a novel inhibitor targeting the beta-catenin/Tcf4 interaction. Biochemistry.

[217] Fang L., Zhu Q., Neuenschwander M., Specker E., Wulf-Goldenberg A., Weis W.I., Von Kries J.P., Birchmeier W. (2016). A small-molecule antagonist of the β-catenin/TCF4 interaction blocks the self-renewal of cancer stem cells and suppresses tumorigenesis. Cancer Res..

[218] Shin S.H., Reddy K., Malakhova M., Liu F., Wang T., Song M., Chen H., Bae K.B., Ryu J., Liu K. (2017). A small molecule inhibitor of the β-catenin-TCF4 interaction suppresses colorectal cancer growth in vitro and in vivo. EBioMedicine.

[219] Emami K.H., Nguyen C., Ma H., Kim D.H., Jeong K.W., Eguchi M., Moon R.T., Teo J.L., Kim H.Y., Moon S.H. (2004). A small molecule inhibitor of beta-catenin/CREB-binding protein transcription [corrected]. Proc. Natl. Acad. Sci. USA.

[220] Gang E.J., Hsieh Y.T., Pham J., Zhao Y., Nguyen C., Huantes S., Park E., Naing K., Klemm L., Swaminathan S. (2014). Small-molecule inhibition of CBP/catenin interactions eliminates drug-resistant clones in acute lymphoblastic leukemia. Oncogene.

[221] Lazarova D.L., Chiaro C., Wong T., Drago E., Rainey A., O’Malley S., Bordonaro M. (2013). CBP Activity Mediates Effects of the Histone Deacetylase Inhibitor Butyrate on WNT Activity and Apoptosis in Colon Cancer Cells. J. Cancer.

[222] Ko A.H., Chiorean E.G., Kwak E.L., Lenz H.-J., Nadler P.I., Wood D.L., Fujimori M., Inada T., Kouji H., McWilliams R.R. (2016). Final results of a phase Ib dose-escalation study of PRI-724, a CBP/beta-catenin modulator, plus gemcitabine (GEM) in patients with advanced pancreatic adenocarcinoma (APC) as second-line therapy after FOLFIRINOX or FOLFOX. J. Clin. Oncol..

[223] Gay D.M., Ridgway R.A., Muller M., Hodder M.C., Hedley A., Clark W., Leach J.D., Jackstadt R., Nixon C., Huels D.J. (2019). Loss of BCL9/9l suppresses Wnt driven tumourigenesis in models that recapitulate human cancer. Nat. Commun..

[224] Mieszczanek J., van Tienen L.M., Ibrahim A.E.K., Winton D.J., Bienz M. (2019). Bcl9 and Pygo synergise downstream of Apc to effect intestinal neoplasia in FAP mouse models. Nat. Commun..

[225] Dale T., Clarke P.A., Esdar C., Waalboer D., Adeniji-Popoola O., Ortiz-Ruiz M.J., Mallinger A., Samant R.S., Czodrowski P., Musil D. (2015). A selective chemical probe for exploring the role of CDK8 and CDK19 in human disease. Nat. Chem. Biol..

[226] Hamanaka N., Nakanishi Y., Mizuno T., Horiguchi-Takei K., Akiyama N., Tanimura H., Hasegawa M., Satoh Y., Tachibana Y., Fujii T. (2019). YES1 Is a Targetable Oncogene in Cancers Harboring YES1 Gene Amplification. Cancer Res..

[227] Chen D.Y., Lee Y., Van Tine B.A., Searleman A.C., Westergard T.D., Liu H., Tu H.C., Takeda S., Dong Y., Piwnica-Worms D.R. (2012). A pharmacologic inhibitor of the protease Taspase1 effectively inhibits breast and brain tumor growth. Cancer Res..

[228] Liu J., Pan S., Hsieh M.H., Ng N., Sun F., Wang T., Kasibhatla S., Schuller A.G., Li A.G., Cheng D. (2013). Targeting Wnt-driven cancer through the inhibition of Porcupine by LGK974. Proc. Natl. Acad. Sci. USA.

[229] Madan B., Ke Z., Harmston N., Ho S.Y., Frois A.O., Alam J., Jeyaraj D.A., Pendharkar V., Ghosh K., Virshup I.H. (2016). Wnt addiction of genetically defined cancers reversed by PORCN inhibition. Oncogene.

[230] Fischer M.M., Cancilla B., Yeung V.P., Cattaruzza F., Chartier C., Murriel C.L., Cain J., Tam R., Cheng C.Y., Evans J.W. (2017). WNT antagonists exhibit unique combinatorial antitumor activity with taxanes by potentiating mitotic cell death. Sci. Adv..

[231] Jimeno A., Gordon M., Chugh R., Messersmith W., Mendelson D., Dupont J., Stagg R., Kapoun A.M., Xu L., Uttamsingh S. (2017). A First-in-Human Phase I Study of the Anticancer Stem Cell Agent Ipafricept (OMP-54F28), a Decoy Receptor for Wnt Ligands, in Patients with Advanced Solid Tumors. Clin. Cancer Res..

[232] Lavergne E., Hendaoui I., Coulouarn C., Ribault C., Leseur J., Eliat P.A., Mebarki S., Corlu A., Clément B., Musso O. (2011). Blocking Wnt signaling by SFRP-like molecules inhibits in vivo cell proliferation and tumor growth in cells carrying active β-catenin. Oncogene.

[233] Canesin G., Evans-Axelsson S., Hellsten R., Krzyzanowska A., Prasad C.P., Bjartell A., Andersson T. (2017). Treatment with the WNT5A-mimicking peptide Foxy-5 effectively reduces the metastatic spread of WNT5A-low prostate cancer cells in an orthotopic mouse model. PLoS ONE.

[234] Giraudet A.L., Cassier P.A., Iwao-Fukukawa C., Garin G., Badel J.N., Kryza D., Chabaud S., Gilles-Afchain L., Clapisson G., Desuzinges C. (2018). A first-in-human study investigating biodistribution, safety and recommended dose of a new radiolabeled MAb targeting FZD10 in metastatic synovial sarcoma patients. BMC Cancer.

[235] Lu W., Lin C., Roberts M.J., Waud W.R., Piazza G.A., Li Y. (2011). Niclosamide suppresses cancer cell growth by inducing Wnt co-receptor LRP6 degradation and inhibiting the Wnt/β-catenin pathway. PLoS ONE.

[236] Lu D., Choi M.Y., Yu J., Castro J.E., Kipps T.J., Carson D.A. (2011). Salinomycin inhibits Wnt signaling and selectively induces apoptosis in chronic lymphocytic leukemia cells. Proc. Natl. Acad. Sci. USA.

[237] Tumova L., Pombinho A.R., Vojtechova M., Stancikova J., Gradl D., Krausova M., Sloncova E., Horazna M., Kriz V., Machonova O. (2014). Monensin inhibits canonical Wnt signaling in human colorectal cancer cells and suppresses tumor growth in multiple intestinal neoplasia mice. Mol. Cancer Ther..

[238] Zhang M., Haughey M., Wang N.Y., Blease K., Kapoun A.M., Couto S., Belka I., Hoey T., Groza M., Hartke J. (2020). Targeting the Wnt signaling pathway through R-spondin 3 identifies an anti-fibrosis treatment strategy for multiple organs. PLoS ONE.

[239] Grandy D., Shan J., Zhang X., Rao S., Akunuru S., Li H., Zhang Y., Alpatov I., Zhang X.A., Lang R.A. (2009). Discovery and characterization of a small molecule inhibitor of the PDZ domain of dishevelled. J. Biol. Chem..

[240] Fujii N., You L., Xu Z., Uematsu K., Shan J., He B., Mikami I., Edmondson L.R., Neale G., Zheng J. (2007). An antagonist of dishevelled protein-protein interaction suppresses beta-catenin-dependent tumor cell growth. Cancer Res..

[241] Shan J., Shi D.L., Wang J., Zheng J. (2005). Identification of a specific inhibitor of the dishevelled PDZ domain. Biochemistry.

[242] Huang S.M., Mishina Y.M., Liu S., Cheung A., Stegmeier F., Michaud G.A., Charlat O., Wiellette E., Zhang Y., Wiessner S. (2009). Tankyrase inhibition stabilizes axin and antagonizes Wnt signalling. Nature.

[243] Narwal M., Venkannagari H., Lehtiö L. (2012). Structural basis of selective inhibition of human tankyrases. J. Med. Chem..

[244] Waaler J., Machon O., Tumova L., Dinh H., Korinek V., Wilson S.R., Paulsen J.E., Pedersen N.M., Eide T.J., Machonova O. (2012). A novel tankyrase inhibitor decreases canonical Wnt signaling in colon carcinoma cells and reduces tumor growth in conditional APC mutant mice. Cancer Res..

[245] Waaler J., Machon O., von Kries J.P., Wilson S.R., Lundenes E., Wedlich D., Gradl D., Paulsen J.E., Machonova O., Dembinski J.L. (2011). Novel synthetic antagonists of canonical Wnt signaling inhibit colorectal cancer cell growth. Cancer Res..

[246] Stratford E.W., Daffinrud J., Munthe E., Castro R., Waaler J., Krauss S., Myklebost O. (2014). The tankyrase-specific inhibitor JW74 affects cell cycle progression and induces apoptosis and differentiation in osteosarcoma cell lines. Cancer Med..

[247] Lau T., Chan E., Callow M., Waaler J., Boggs J., Blake R.A., Magnuson S., Sambrone A., Schutten M., Firestein R. (2013). A novel tankyrase small-molecule inhibitor suppresses APC mutation-driven colorectal tumor growth. Cancer Res..

[248] Menon M., Elliott R., Bowers L., Balan N., Rafiq R., Costa-Cabral S., Munkonge F., Trinidade I., Porter R., Campbell A.D. (2019). A novel tankyrase inhibitor, MSC2504877, enhances the effects of clinical CDK4/6 inhibitors. Sci. Rep..

[249] Mizutani A., Yashiroda Y., Muramatsu Y., Yoshida H., Chikada T., Tsumura T., Okue M., Shirai F., Fukami T., Yoshida M. (2018). RK-287107, a potent and specific tankyrase inhibitor, blocks colorectal cancer cell growth in a preclinical model. Cancer Sci..

[250] Arqués O., Chicote I., Puig I., Tenbaum S.P., Argilés G., Dienstmann R., Fernández N., Caratù G., Matito J., Silberschmidt D. (2016). Tankyrase Inhibition Blocks Wnt/β-Catenin Pathway and Reverts Resistance to PI3K and AKT Inhibitors in the Treatment of Colorectal Cancer. Clin. Cancer Res..

[251] Thorne C.A., Hanson A.J., Schneider J., Tahinci E., Orton D., Cselenyi C.S., Jernigan K.K., Meyers K.C., Hang B.I., Waterson A.G. (2010). Small-molecule inhibition of Wnt signaling through activation of casein kinase 1α. Nat. Chem. Biol..

[252] Atkinson J.M., Rank K.B., Zeng Y., Capen A., Yadav V., Manro J.R., Engler T.A., Chedid M. (2015). Activating the Wnt/β-Catenin Pathway for the Treatment of Melanoma--Application of LY2090314, a Novel Selective Inhibitor of Glycogen Synthase Kinase-3. PLoS ONE.

[253] Hwang S.Y., Deng X., Byun S., Lee C., Lee S.J., Suh H., Zhang J., Kang Q., Zhang T., Westover K.D. (2016). Direct Targeting of β-Catenin by a Small Molecule Stimulates Proteasomal Degradation and Suppresses Oncogenic Wnt/β-Catenin Signaling. Cell Rep..

[254] Kimura K., Ikoma A., Shibakawa M., Shimoda S., Harada K., Saio M., Imamura J., Osawa Y., Kimura M., Nishikawa K. (2017). Safety, Tolerability, and Preliminary Efficacy of the Anti-Fibrotic Small Molecule PRI-724, a CBP/β-Catenin Inhibitor, in Patients with Hepatitis C Virus-related Cirrhosis: A Single-Center, Open-Label, Dose Escalation Phase 1 Trial. EBioMedicine.

[255] Takada K., Zhu D., Bird G.H., Sukhdeo K., Zhao J.J., Mani M., Lemieux M., Carrasco D.E., Ryan J., Horst D. (2012). Targeted disruption of the BCL9/β-catenin complex inhibits oncogenic Wnt signaling. Sci. Transl. Med..

[256] Feng M., Jin J.Q., Xia L., Xiao T., Mei S., Wang X., Huang X., Chen J., Liu M., Chen C. (2019). Pharmacological inhibition of β-catenin/BCL9 interaction overcomes resistance to immune checkpoint blockades by modulating T(reg) cells. Sci. Adv..

[257] Masuda M., Sawa M., Yamada T. (2015). Therapeutic targets in the Wnt signaling pathway: Feasibility of targeting TNIK in colorectal cancer. Pharmacol. Ther..

